# Explaining Performance on Interval and Ratio Schedules with a Molar View of Behavior

**DOI:** 10.1007/s40614-025-00455-3

**Published:** 2025-05-21

**Authors:** William M. Baum

**Affiliations:** University of California, Davis, and University of New Hampshire, 1305 Boulevard Way, Apt. 302, Walnut Creek, CA 94595 USA

**Keywords:** Ratio schedules, Interval schedules, Phylogenetically important event, Induction, Feedback function, Feedback system, Pigeons

## Abstract

Some of the most basic phenomena in behavior analysis are the differences between performance on interval and ratio schedules. They have long been known and long puzzled over. Previous attempts to explain the performances have foundered either because they lacked a mechanism or because they adopted a molecular view of behavior based on discrete responses and contiguity. The molar view of behavior offers the sought-for explanation of differences in activity rate and the inability of ratio schedules to maintain activity at low food rates. The present account relies on induction by phylogenetically important events (PIE) according to power functions, molar feedback functions, and the framework of matching theory. A model described by a feedback system with all parameters the same predicts the relations between activity rate and PIE rate. The difference in overall activity rate arises from a difference in units of activities selected by ratio and interval schedules. The results demonstrate the greater explanatory power of the molar view of behavior.

This article reports a theoretical model that solves a long-unsolved problem: explaining performances on variable-interval (VI) schedules and variable-ratio (VR) schedules. Where other attempts at explanation have relied on the traditional molecular view, based on discrete responses, response-reinforcer contiguity, and reinforcement, and failed, this theory succeeds by adopting a molar view of behavior to illuminate these basic phenomena (Baum, 2002, 2018a). I begin by briefly explaining the molar view and then apply it to VI and VR schedules.

In an experiment on operant or instrumental behavior, an organism such as a rat, pigeon, monkey, or human interacts with a device such as a lever, key, or button attached to a switch. Researchers commonly count operations of the switch to measure the amount of interaction with the device. No account is usually taken of exactly how the switch was operated—whether one body part or another. In a throwback to the days of studying reflexes, researchers call each switch operation a “response” and the number of operations divided by the time interval in which they occurred “response rate” (Skinner, 1938).

Baum and Rachlin (1969), reporting on an experiment on time allocation, pointed out that time is the universal measure of behavior and argued that if one considered each switch operation to indicate a certain amount of time spent in an activity (e.g., key pecking) response rate converts to proportion of time spent in the activity out of the time available. Later studies confirmed the equivalence between “responses” and time for lever pressing (Baum, 1975, 1976).

We can reconcile experiments reporting response rates with those reporting time spent by regarding both as measures of *activity rate*. When the measure of time is proportion of time spent in an activity out of the available time or the number of switch operations in the available time, we may call either measure activity rate.

A switch operation occasionally produces an event such as a prey item, a bit of food, a drink of water, or a potential mate—events belonging to a larger category that includes also predators, shelter, and injury—a *phylogenetically important event* (PIE; Baum, 2012, 2024) or “statistical fitness predictor” (Borgstede & Eggert, 2021). These events have powerful effects on behavior, because organisms have a long evolutionary history with them. To say an event is “phylogenetically important” is to say it affects the likelihood of survival and reproduction—what is called “fitness” in evolutionary theory. Such an event induces whole patterns of activity, including both further operant activity and some nonoperant activity too, and these activities compete with each other to a lesser or greater extent (Baum & Aparicio, 2020; Breland & Breland, 1961; Segal, 1972; Staddon, 1977).

Broadly speaking, when inducing events (PIEs) are contingent on operant activity, they may be scheduled in two ways: (1) either the inducer (e.g., food) is earned for activity alone; or (2) the inducer requires only a bit of activity at the end of a period of time. The former is called a ratio schedule and the latter is called an interval schedule. If each earned inducer requires a fixed amount of activity, the schedule is a fixed-ratio (FR) schedule. If inducers require a variable, unpredictable, amount of activity, the schedule is a variable-ratio (VR) schedule. Likewise, if each inducer is available only after a fixed time interval, the schedule is a fixed-interval (FI) schedule, and if inducers are available only after variable, unpredictable, intervals, the schedule is a variable-interval (VI) schedule (Ferster & Skinner, 1957). Natural-world VR schedules might be hunting and gambling: the more time spent in the activity, the more earnings (prey or payoffs), but the earnings are unpredictable. An example of a VI schedule might be receiving an expected email: one can only wait and check.

Since the 1950s researchers have known three basic differences between the performances generated by ratio and interval schedules (Ferster & Skinner, 1957). First, ratio schedules maintain extremely high activity rates, whereas interval schedules maintain lower, moderate activity rates. Second, interval schedules maintain higher and lower activity rates as the schedule-provided PIE rate goes from high to low. Third, interval schedules maintain activity no matter how low the PIE rate goes. In contrast, ratio schedules maintain activity only when the PIE rate is high. As the ratio requirement increases, activity becomes increasingly erratic, and activity rate drops to zero when the ratio requirement is too high—a phenomenon called “ratio strain” (Ferster & Skinner, 1957).

FI and FR schedules generate pauses before activity begins and approaches the work or time required to produce an inducer (e.g., food delivery). The duration of the pause varies directly with the FI or FR. “Pause” means other activities occurring instead of the operant activity (Staddon, 1977). Both the FI pause and FR pause result from activities induced by the just-presented food that must occur before they are excluded from the concentrated activity that completes the FI or FR requirement. When activity begins following the pause, the activity rate on the FI resembles that on a VI, and the activity rate on the FR resembles that on a VR (Felton & Lyon, 1966; Schneider, 1969). Thus, the key challenge is explaining performance on VI and VR schedules.

Figure 1 illustrates the differences between VR and VI schedules as captured by their feedback functions, the functions specifying the dependence of PIE rate on activity rate prescribed by the different schedules. The feedback function for a VR schedule is a straight line, because the PIE rate (*R*) produced is directly proportional to the activity rate (*B*). The constant of proportionality equals 1/VR, and the equation of the line is: $R=\frac{B}{VR}$. The top panel shows feedback lines for four VR schedules. The steeper the slope, the richer the schedule. The lines marked a, b, and c all maintain high activity rates, even though the rates of producing PIEs decrease as the slopes decrease. A threshold line (broken line) defines a slope below which activity ceases, where ratio strain predominates. It marks the cutoff in VR activity. Thus, the line marked d fails to maintain any activity; activity rate falls to 0.

**Fig. 1:**
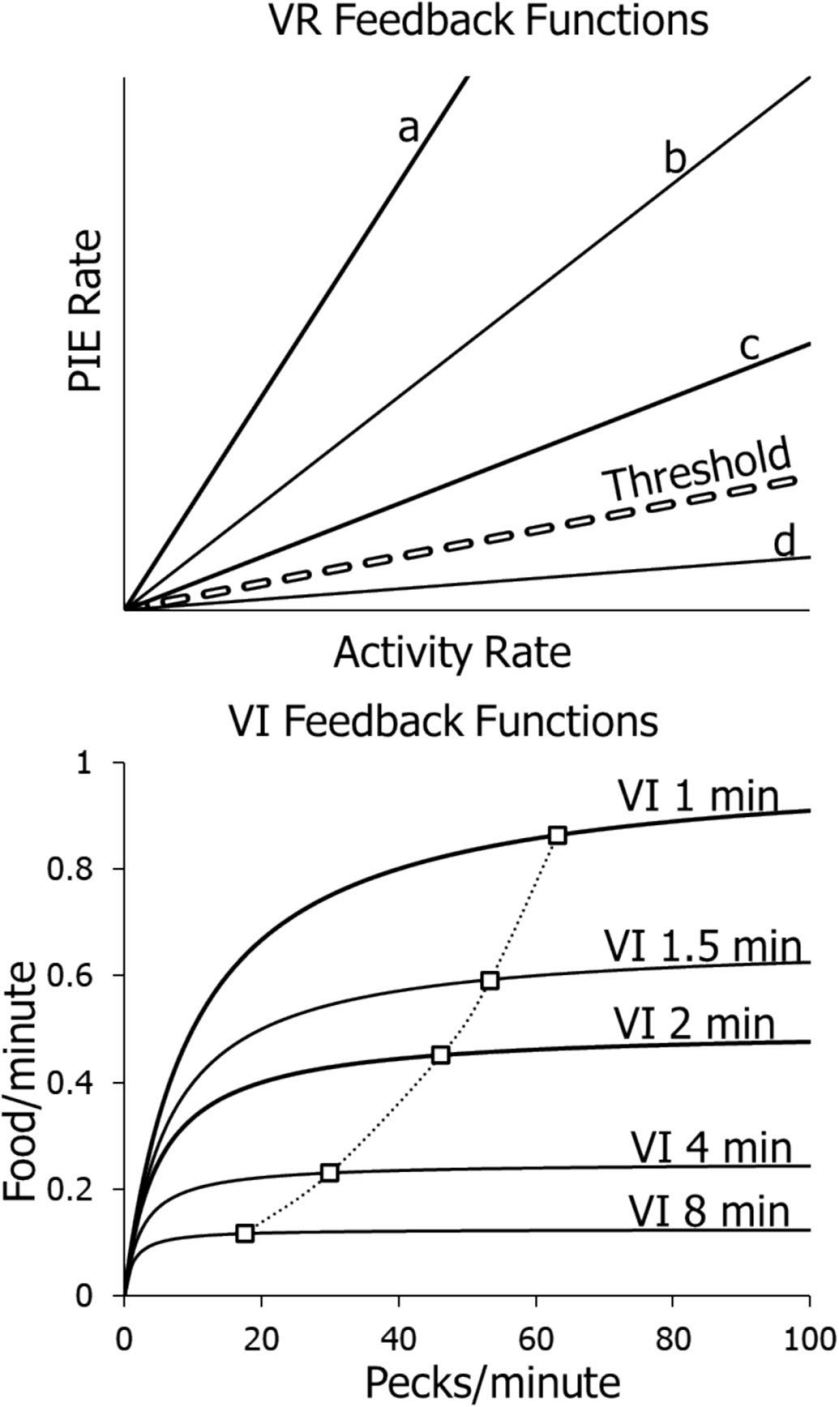
Feedback Functions for VR and VI Schedules. *Note.* **Top**: VR feedback lines a, b, and c maintain high activity rates, the steeper the slope the higher the rate. Line d lies below the threshold line (dashed) and maintains no operant activity. **Bottom**: Several VI feedback curves, all of which maintain activity, as illustrated by the typical performances shown (squares)

The bottom panel in Fig. 1 shows five VI feedback functions. Each curve begins at the origin and increases toward approaching an asymptote set by the scheduled rate. The curve is steep at the low end of activity rates and flattens for high activity rates. The equation of the VI feedback functions shown is:

1 $$R=\frac{1}{t+\frac{a}{B}},$$

where *t* is the average interval (VI), and *a* is a parameter that accommodates the degree to which activity is organized into bouts and pauses (Gilbert, 1958; Shull et al., 2001). If *a* equals 1.0, switch operations are approximately random and accord with a Poisson process. If activity occurs in bouts, *a* will exceed 1.0 (Baum, 1992). The curves represent feedback when the activity is a pigeon’s pecking at a key. Researchers commonly call each switch operation a “peck.” The inducer or PIE is food, and *R* represents food rate. The squares show the typical stable activity and food rates that the schedules maintain. The dotted curve connects the squares to show a typical result when each of several VI schedules remains in effect until performance stabilizes (e.g., Baum & Grace, 2020; Herrnstein, 1970).

Many researchers have attempted to explain the difference between performances maintained by ratio and interval schedules. The earliest attempt appealed to differential reinforcement of pausing on interval schedules, because the longer the pause before a switch operation, the higher the probability the switch operation will produce food. This theory proved inadequate, however, because it failed to explain the high activity rates on ratio schedules and made the false prediction of extremely low activity rates on interval schedules (Baum, 2018b; Morse, 1966).

Some researchers tried to explain the rate difference by showing that the difference agrees with an optimality model in which activity maximizes food rate or activity maximizes a benefit-to-cost ratio (Baum, 1981; Rachlin, 1978; Staddon, 1980). Optimality accounts in general suffer from the problem that they fail to provide a mechanism by which maximizing comes about. One might imagine some kind of selection, but such a mechanism needs to be spelled out and would not necessarily lead to optimality. A successful account requires a mechanism.

Other researchers approached the problem with a molecular view of behavior, instead of the molar view adopted here (Baum, 2002). The proposed models succeeded to some extent with the straight lines of VR schedules, but failed to account adequately for performance on VI schedules. For example, Perez and Dickinson (2020) tried to model momentary food rates on a VI, from which they concluded that food rate is independent of activity rate, because the VI feedback function does not fit with a molecular view. Gallistel et al. (2019) also assumed that food rate is independent of activity rate on a VI, and also failed as a result to adequately account for the sort of VI performances shown in Fig. 1. McDowell and Kessel (1979) tried to bypass the feedback function and derive a performance function directly, but in doing so assumed no dependency of PIE rate on activity rate. Killeen (2023), although assuming a feedback function like Eq. 1 with *a* equal to 1.0, was able only to fit the average across pigeons of performance at high food rates (Baum, 1993), and not the sort of performances shown in Fig. 1 (bottom). None of these theories predicted the cutoff of activity on lean ratio schedules, and none of them was sufficiently well-developed as to predict the shape of the curves relating activity rate to food rate across VI and VR schedules. None of them offered a single model that explained activity rates on both VI and VR schedules.

Baum and Grace (2020) studied pigeons’ performances on a wide range of VI schedules. Each daily session consisted of seven different components, each with a different schedule. Several different mixed schedules like this remained in effect until performance on all of them appeared stable. The experiment revealed the shifts in performance from the low range of food rates to higher food rates, and finally to the highest possible, with FR 1. We offered a model to explain the overall pattern, but only for VI schedules, because we did no systematic study of VR schedules.

## Theoretical Framework

Earlier papers proposed a conceptual framework for thinking about behavior composed of three laws: (1) Law of Allocation; (2) Law of Induction; and (3) Law of Covariance (Baum, 2018a, 2018b). The following equations express the Law of Allocation:

1a $$\frac{{T}_{j}}{\sum_{i=1}^{N}{T}_{i}}=\frac{{V}_{j}}{\sum_{i=1}^{N}{V}_{i}}$$

1b $$\frac{{B}_{j}}{\sum_{i=1}^{N}{B}_{i}}=\frac{{V}_{j}}{\sum_{i=1}^{N}{V}_{i}}$$

where *T*_*i*_ represents the time taken by Activity *i*, *B*_*i*_ represents the time taken by Activity *i* measured in operations of a key or lever, and *V*_*i*_ represents the competitive weight of Activity *i*, which depends on variables like rate and amount of food, according to the Law of Induction, expressed in the following equation:

2 $${V}_{i}=\prod_{j=1}^{m}{c}_{j}{x}_{ij}^{{s}_{j}}$$

where *x*_*j*_ is a variable like rate, amount, or immediacy, and competitive weight equals the product of *m* power functions with coefficient *c*_*j*_ and exponent *S*_*j*_. The present discussion focuses on food rate, *r*, and the Law of Induction becomes:

3 $${V}_{i}={c}_{i}{r}_{i}^{{S}_{i}}.$$

Using Eq. 3, we may rewrite Eq. 1b to:

4 $$\frac{{B}_{1}}{{B}_{1}+{B}_{0}+{B}_{N}}=\frac{{c}_{1}{r}^{{S}_{1}}}{{c}_{1}{r}^{{S}_{1}}+{c}_{0}{r}^{{S}_{0}}+{V}_{N}},$$

where *B*_*1*_ represents activity rate (e.g., key-peck rate), *B*_*0*_ represents other activities induced by the food, *B*_*N*_ represents activities unrelated to food (e.g., resting or grooming), and *V*_*N*_ represents effects of *B*_*N*_. Two simplifications of Eq. 4 are possible. First, because *B*_*1*_ + *B*_*0*_ + *B*_*N*_ take up all the time available, we may set their sum equal to a constant *K*. Second, when *B*_*1*_ and *B*_*0*_ are large, *B*_*N*_ is relatively small, and *V*_*N*_ may be considered negligible. Thus, we rewrite Eq. 4:

5 $${B}_{1}=\frac{K{V}_{1}}{{V}_{1}+{V}_{0}}=\frac{K{c}_{1}{r}^{{S}_{1}}}{{c}_{1}{r}^{{S}_{1}}+{c}_{0}{r}^{{S}_{0}}},$$

and this equation simplifies to:

6 $${B}_{1}=\frac{K}{1+c{r}^{-S}},$$

where $c=\frac{{c}_{0}}{{c}_{1}}$ and $S={S}_{1}-{S}_{0}$. Equation 6 serves to fit most activity rates. The dotted curve in Fig. 1 represents Eq. 6.

**Fig. 2:**
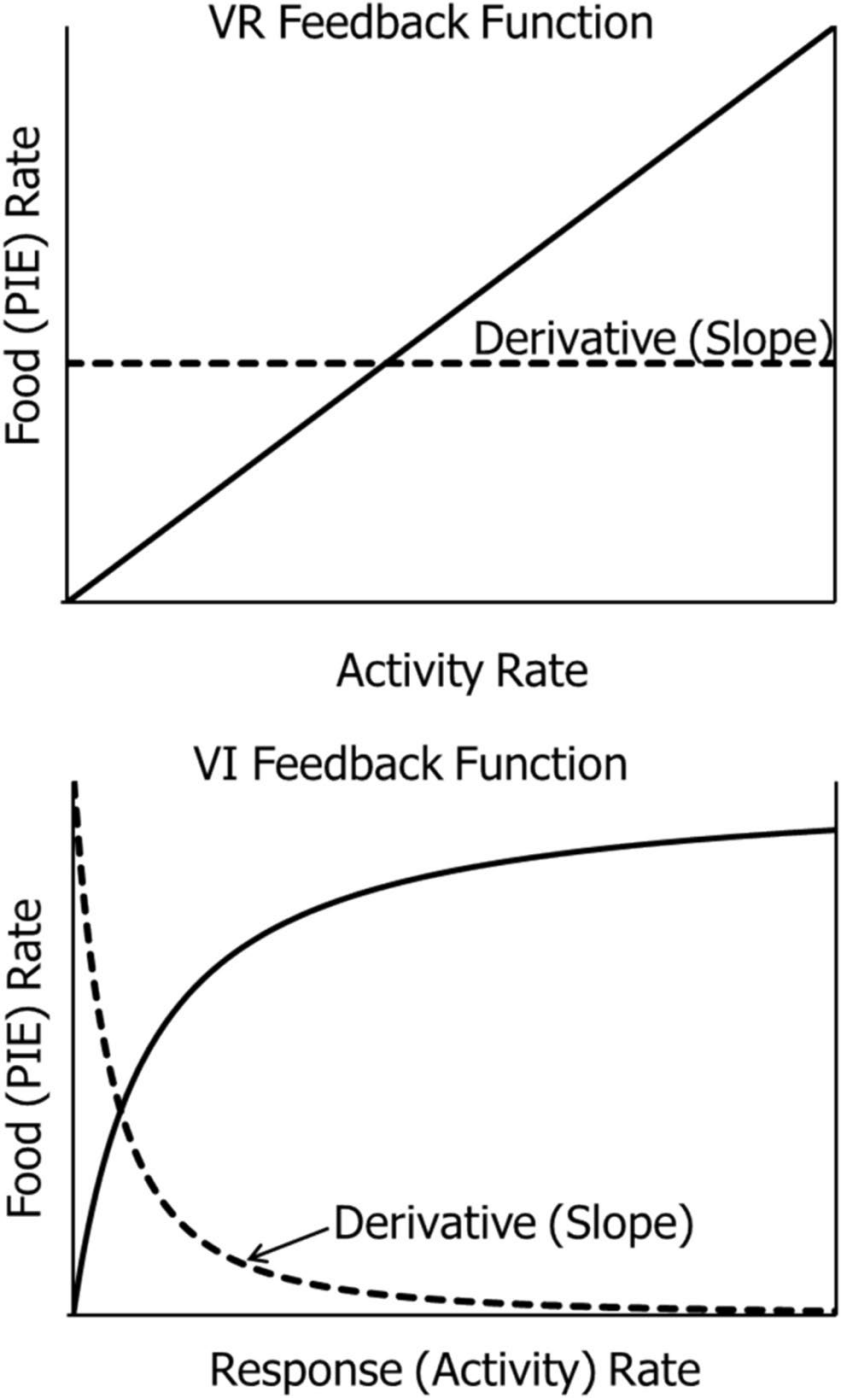
Feedback Functions for VR and VI Schedules with Their Derivatives. *Note.* **Top**: the derivative of a VR line is a constant, shown by the dashed horizontal line. **Bottom**: the derivative (slope) of a VI feedback curve starts high and decreases as activity rate increases

The third law, the Law of Covariance, has two parts, one applying to a stimulus in covariance with a PIE, and the other applying to an activity in covariance with a PIE. The stimulus in covariance with the PIE becomes a conditional inducer. The activity in covariance with the PIE becomes operant activity. This second part of the law applies in the present context. Here covariance refers to $\frac{dR(B)}{dB}$, the derivative or slope of the feedback function, *R(B)*, relating food rate to activity rate, as illustrated in Fig. 2.

The top panel in Fig. 2 shows a feedback function for a VR schedule, the solid line. The equation appears beside it, and the dashed line shows the invariant slope and derivative, which equals 1/VR. The bottom panel shows a feedback function for a VI schedule, the solid curve. It represents Eq. 1. The derivative of this equation appears as a dashed curve showing the steep slope at low activity rates and lower slope at high activity rates.

The Law of Covariance, for the present purpose, is:

7 $$\Delta R=\frac{dR(B)}{dB}\Delta B,$$

which states that any change in activity, *ΔB*, results in a change in PIE rate, *ΔR*, proportional to the derivative of the feedback function, $\frac{dR(B)}{dB}$, where *R(B)* represents the feedback function. Equation 7 shows the mechanism by which PIE (food) rate adjusts as activity rate varies. It is key to describing the behavior-environment feedback system, as we shall see.

The two feedback functions in Fig. 2 contrast in that one is a straight line and the other is a curve approaching an upper limit or asymptote. The derivatives contrast accordingly. One is invariant, for the VR, and the other, for the VI, starts high at low activity rates and descends as activity rate increases, approaching zero. Equation 7 for VR means that as *B* increases or decreases, *R* always increases or decreases proportionally with *B*. Equation 7 for VI means that as *B* increases or decreases, *R* increases or decreases steeply at low activity rates, but less so for higher activity rates, and not at all for the highest activity rates. For VR, selection invariably favors either higher or lower activity rates, whereas for VI, selection is strong at low activity rates and weak at high activity rates.

## Stable Performances on VI and VR Schedules

The most parametric data available showing performance on VI and VR schedules come from experiments with pigeons receiving food. Thus, we will focus on experiments with pigeons earning food by operating keys.

**Fig. 3:**
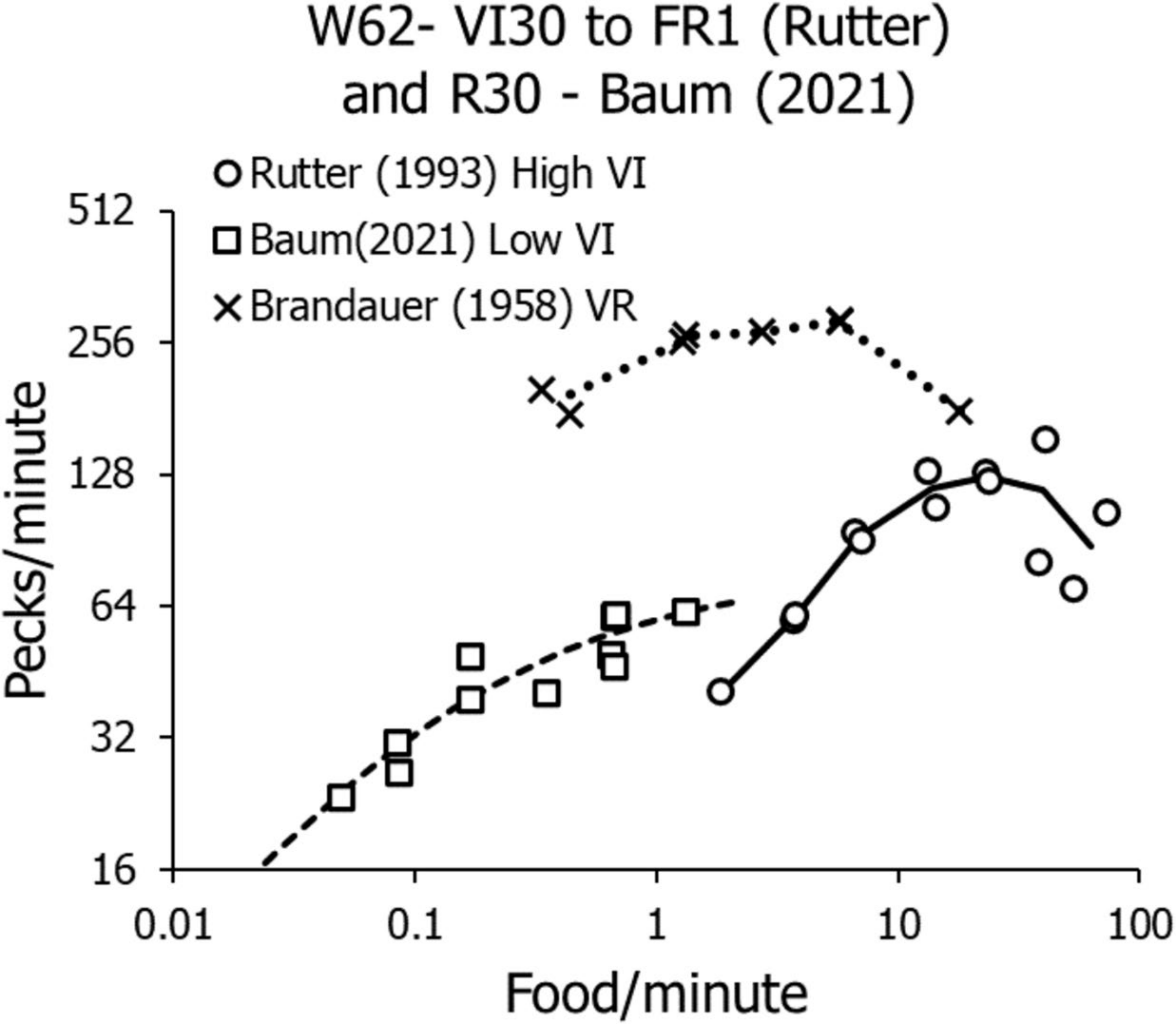
Performances of Individual Pigeons on a Range of VI and VR Schedules. *Note.* Squares show data gathered by Baum (2021; Pigeon R30). Circles show data gathered by S. Rutter (Baum, 1993; Pigeon W62). Xs show data from Brandauer’s (1958) doctoral dissertation (Pigeon 15)

Figure 3 shows results from three different experiments. The squares show stable activity rates of a pigeon in the relatively low range of food rates up to about five deliveries per minute (Baum, 2021). The dashed curve shows the fit of Eq. 6 to the squares. It resembles the curve shown in Fig. 2. The circles show the stable performances of a pigeon exposed to relatively high food rates from 1.8 up to 63 (on FR 1) deliveries per minute. The solid lines connect means across replications. These data were gathered by Sandra Rutter (in Baum, 1993). The Xs show stable activity rates on a range of VR schedules. They are from a PhD dissertation by Brandauer (1958). The dotted lines connect means of replications.

Figure 3 shows five phenomena that require explanation. First, in the low range of VI food rates, peck rates conform to a well-known concave-downward pattern that is well-captured by Eq. 6 (Baum & Grace, 2020; deVilliers, 1977; Herrnstein, 1970). Second, as VI food rate rises above about 3–5 food/min, peck rate passes through an inflection point and rises in a concave-upward pattern as food rate increases. Third, VR schedules maintain much higher peck rates than VI schedules overall (Ferster & Skinner, 1957). Fourth, VR schedules maintain pecking only down to food rates as low as about 0.4 food/min, and lesser food rates fail to maintain any activity. Fifth, both VI and VR peck rates tend to decline at the very highest food rates.

**Fig. 4:**
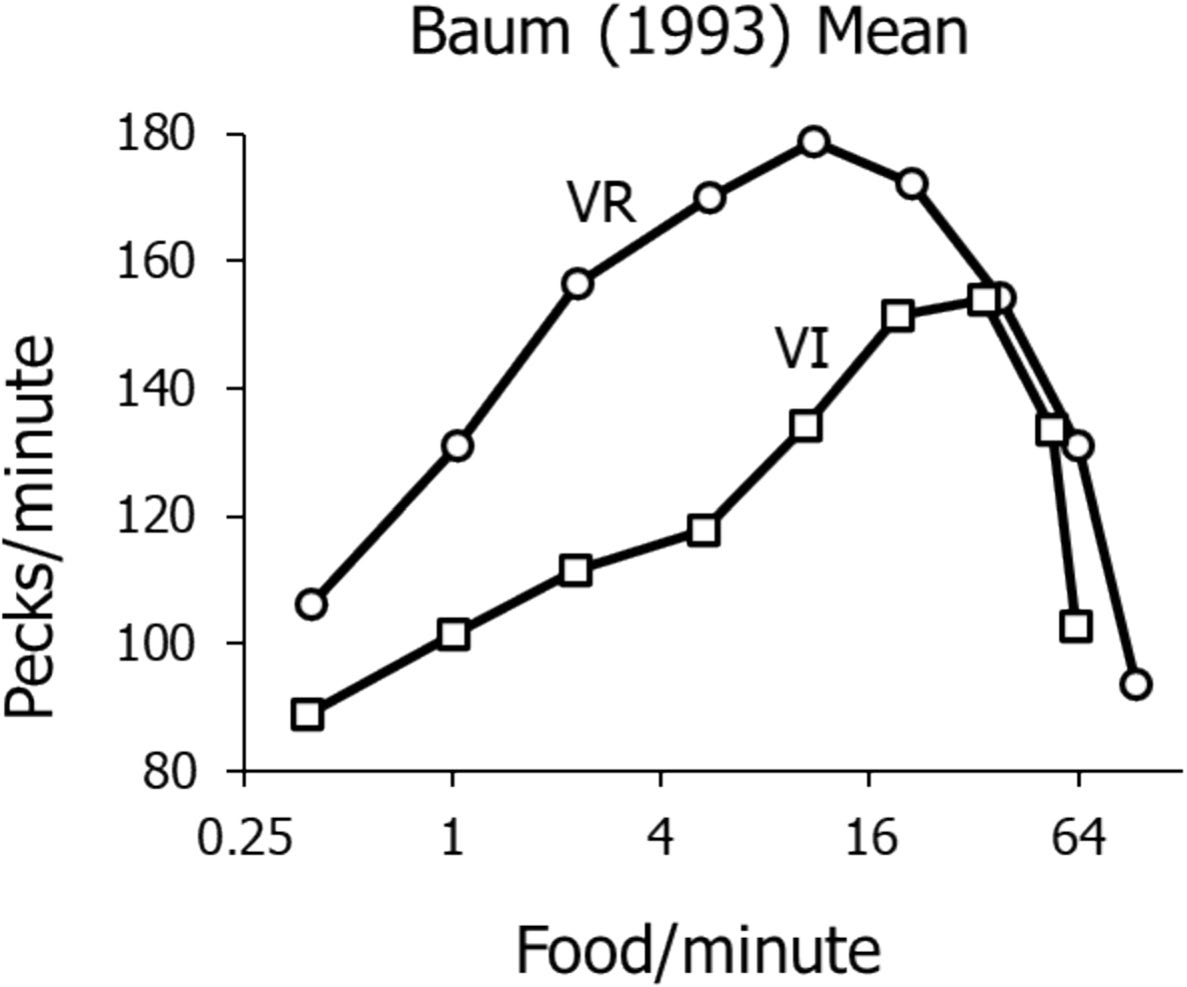
Activity Rates in a Multiple Schedule in which the VI Component was Yoked to the VR Component to Approximately Match the Food Rates

Although Fig. 3 illustrates these five phenomena, it does so with data from three different experiments. Seeing them all in data from individual pigeons would be preferable, because it would eliminate any doubt that any of the phenomena were peculiar to a particular experiment. Figure 4 shows such data.

Figure 4 shows data from an experiment in which a VR schedule alternated with a VI schedule in a multiple-schedule arrangement in which the intervals generated in the VR component were played back in the yoked VI component to approximately match the food rates in the two components. Components were widely separated by blackouts between components that lasted a minimum of 2 min. The VR was varied across conditions that were continued until performance stabilized. The food rate was varied across conditions over the widest possible range, to FR 1 at the extreme high end and down to the lowest rate (largest VR) that would sustain pecking. The data shown are means across four pigeons, but are representative of the individual performances (Baum, 1993).

The low range of food rates in Fig. 4 was curtailed by the VR cutoff at low rates (Fig. 1). Hence only 4 points appear in that range, representing the relatively flat part of the low-range rates shown in Fig. 3 (squares). Above about 5 food/min, peck rate begins climbing again, up to a high close to the peck rate maintained by the rich VR schedules. At the very highest food rates, both VI and VR peck rates fall off.

**Fig. 5:**
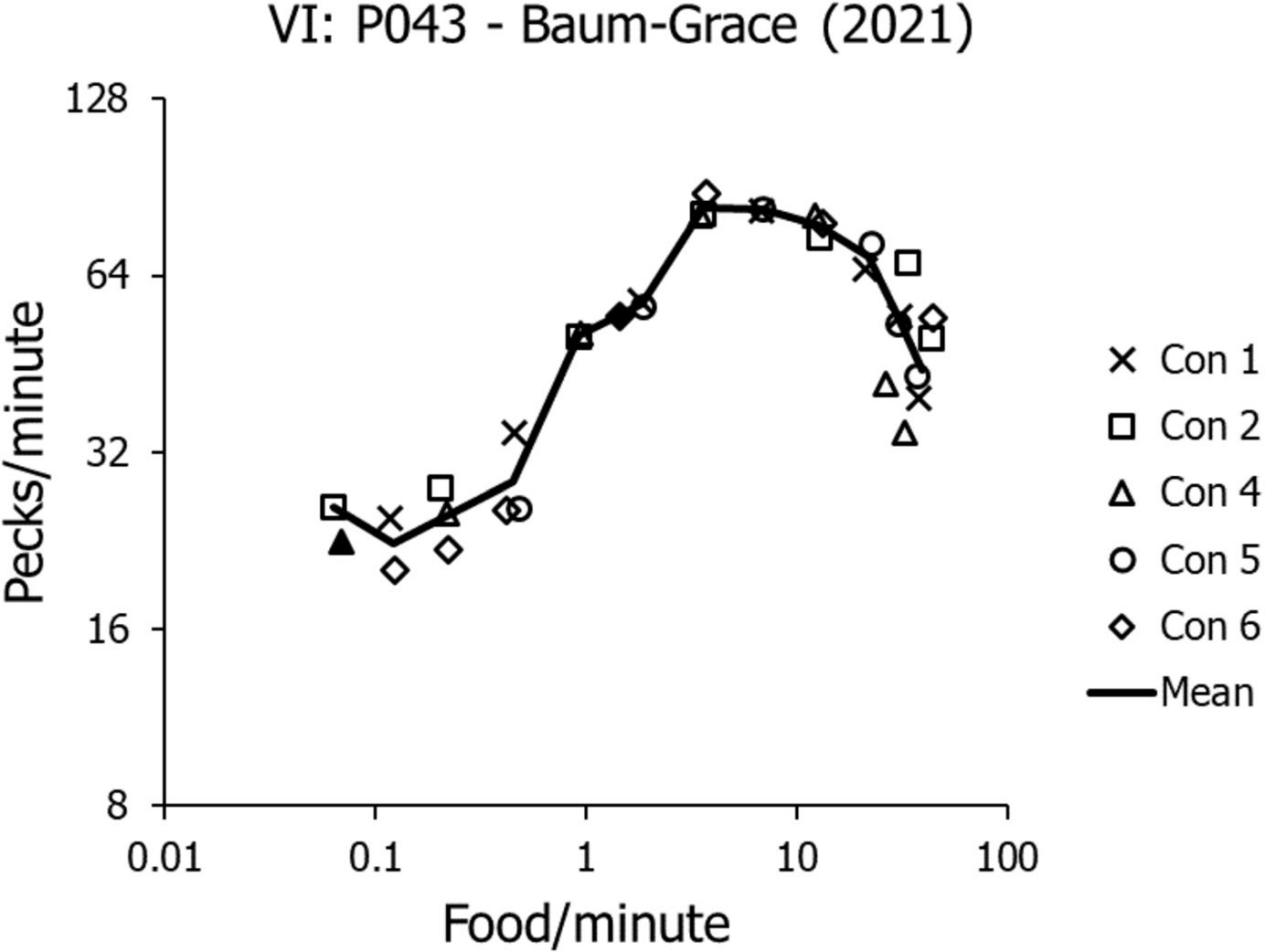
Activity Rates across a Wide Range of Food Rates for Pigeon P043 in an Experiment by Baum and Grace (2020). *Note.* The solid line shows means across six different conditions

Figure 5 shows the VI performance pattern from one pigeon in another experiment (Baum & Grace, 2020). The pigeon was exposed to 7 VI schedules within each session of each condition. Six different conditions were maintained for enough sessions to produce stable performance in all components. The VI schedules ranged from VI 1200 s to VI 1 s and FR 1. No discriminative stimuli accompanied components, but peck rate stabilized within components, and Fig. 5 shows these stable peck rates. The solid line connects means across replications of schedules. The same pattern appears as in Fig. 4: flat in the low range, changing to an increasing trend at a certain food rate (about 0.1 food/min here), then rising to a maximum with increasing food rate, and finally decreasing at the highest food rates.

**Fig. 6:**
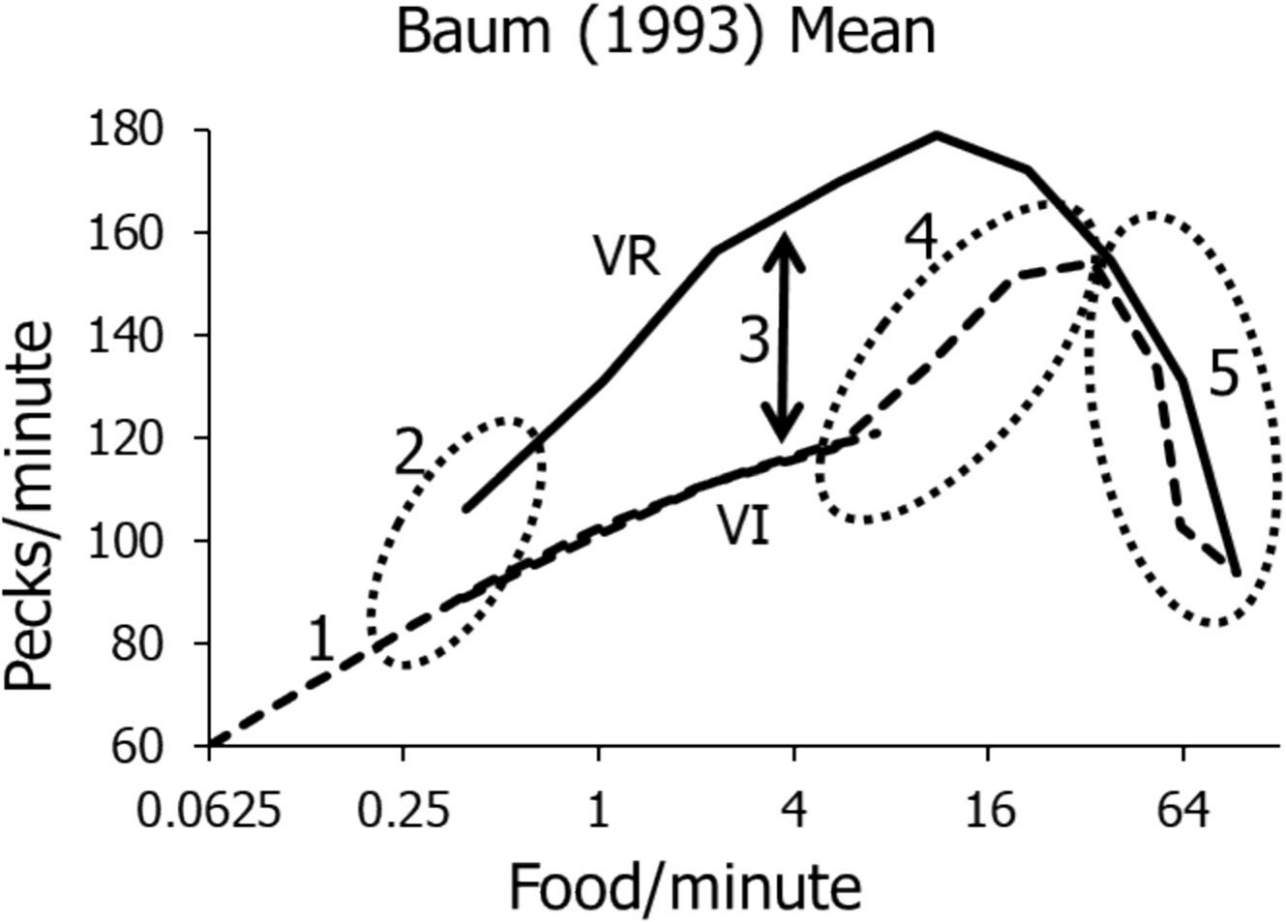
Five Phenomena of VI and VR Schedules. *Note.* (1) dashed curve, VI rates at relatively low food rates; (2) failure of VR schedules (solid lines) to maintain activity below food rates still supporting activity by VI schedules; (3) arrow showing higher activity rates maintained by VR schedules than VI schedules at the same food rate; (4) upturn in activity rates maintained by VI schedules at relatively high food rates. (5) downturn at the highest food rates for both VI and VR

Figure 6 illustrates the five phenomena of VI and VR performances that require explanation: (1) the low-range VI peck rates fitted by Eq. 6; (2) the inability of VR schedules to maintain activity at low food rates compared with VI schedules’ maintaining activity at extremely low food rates; (3) the canonical difference in peck rate between VR and VI performance in the midrange of food rates; (4) the upturn in VI peck rates in the midrange of food rates; and (5) the downturn in peck rate at the highest food rates.

## The High-Rate Downturn

**Fig. 7:**
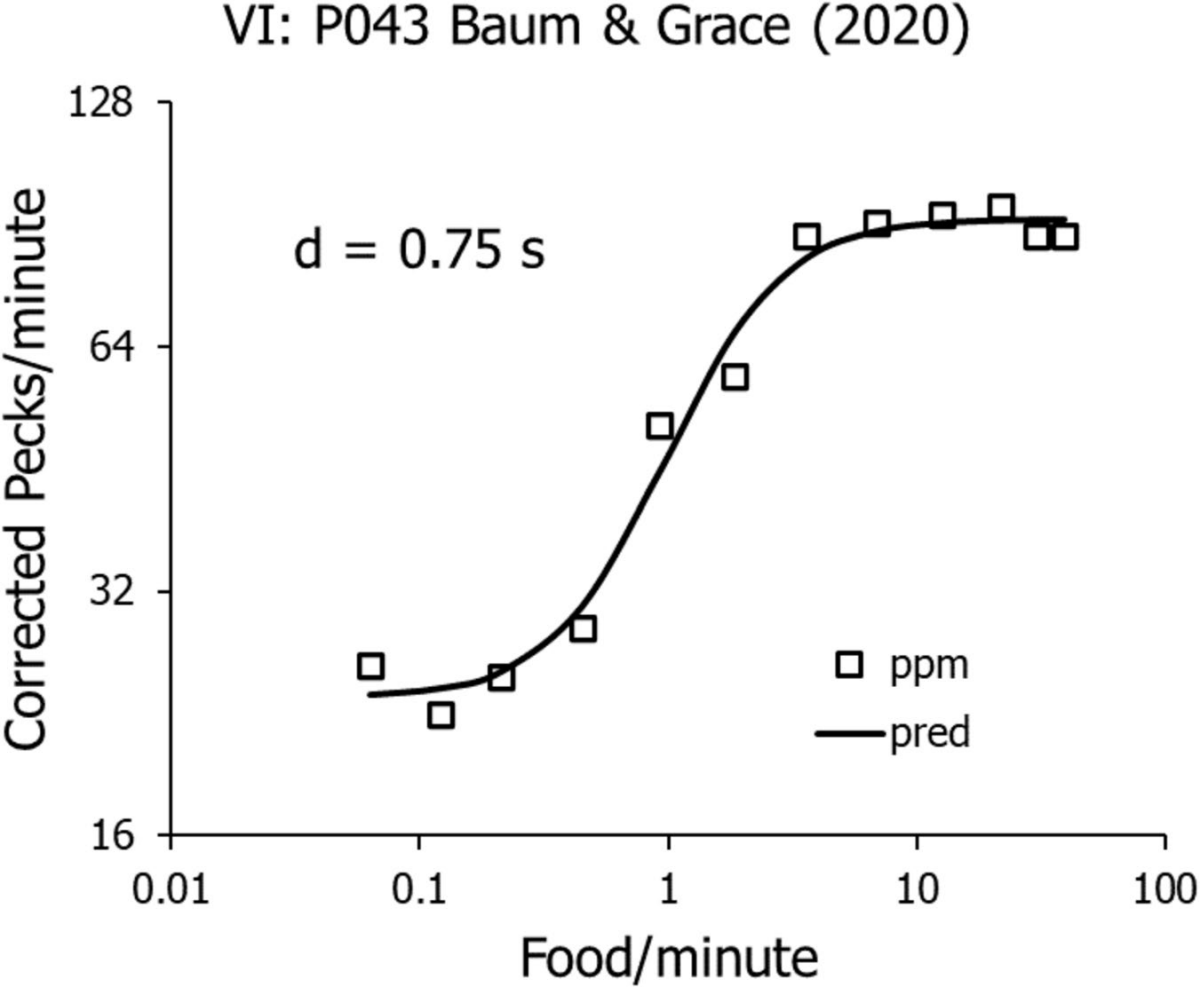
Mean Peck Rates Shown in Fig. 6 Corrected According to Eq. 8. *Note.* The curve is fitted according to the model derived by Baum and Grace (2020)

Of these five, the easiest one to explain is the downturn at extreme food rates. For these experiments with pigeons, the downturn is an artifact of the apparatus. For a pigeon to withdraw its head from the food hopper and raise it up to peck at the response key requires a small amount of time. Likewise, for a rat to move from the food hopper back to the lever in the standard arrangement requires a small amount of time (Baum & Davison, 2014; Fig. 10). At extremely high food rates, when food is arriving about once a second, a fraction of a second suffices to lower the recorded activity (peck) rate. The small delay only matters at the highest food rates; at lower food rates, it is negligible. The simplest way to deal with this artifact is to correct the recorded peck rate according to the equation:

8 $$B=\frac{{B}^{\prime}}{1+dR},$$

where *B’* is recorded peck rate, *R* is food rate, and *d* is the estimated time for the pigeon to move from hopper to key. The parameter *d* is typically less than a second. For example, the rates shown in Figs. 4 and 6 are corrected to be flat at high food rates with *d* equal to 0.5 s (Baum, 1993). Figure 7 shows the means from Fig. 5 (Baum & Grace, 2020) corrected with *d* equal to 0.75 s. The fitted curve shows a model that Baum and Grace fitted to the data that accounted for the upturn in the VI peck rates; the full explanation may be found there.

**Fig. 8:**
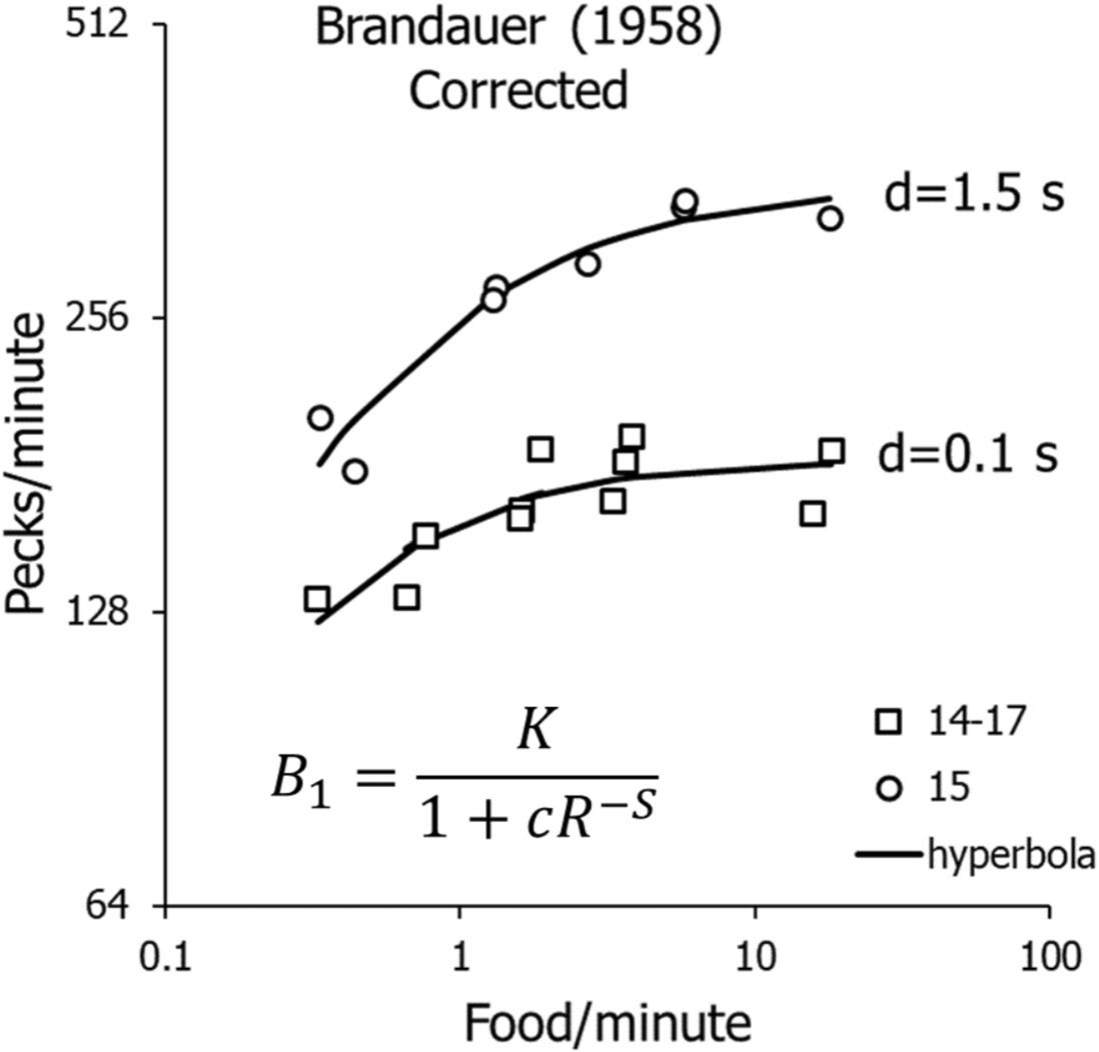
Brandauer’s (1958) VR Activity Rates Corrected According to Eq. 8. *Note.* Equation 6 (shown) was fitted to Pigeon 15’s data. The squares represent activity rates of Pigeons 14 and 17 and were fitted together because they were indistinguishable

Figure 8 shows Brandauer’s (1958) VR performances corrected. The lower set, combining Pigeons 14 and 17, required *d* equal to 0.1 s, and the other (Pigeon 15) required *d* equal to 1.5 s. The curves show fits of Eq. 6. Because the high-rate downturn is explained and corrected by Eq. 8, we will now address only corrected peck rates.

## The VI Upturn

**Fig. 9:**
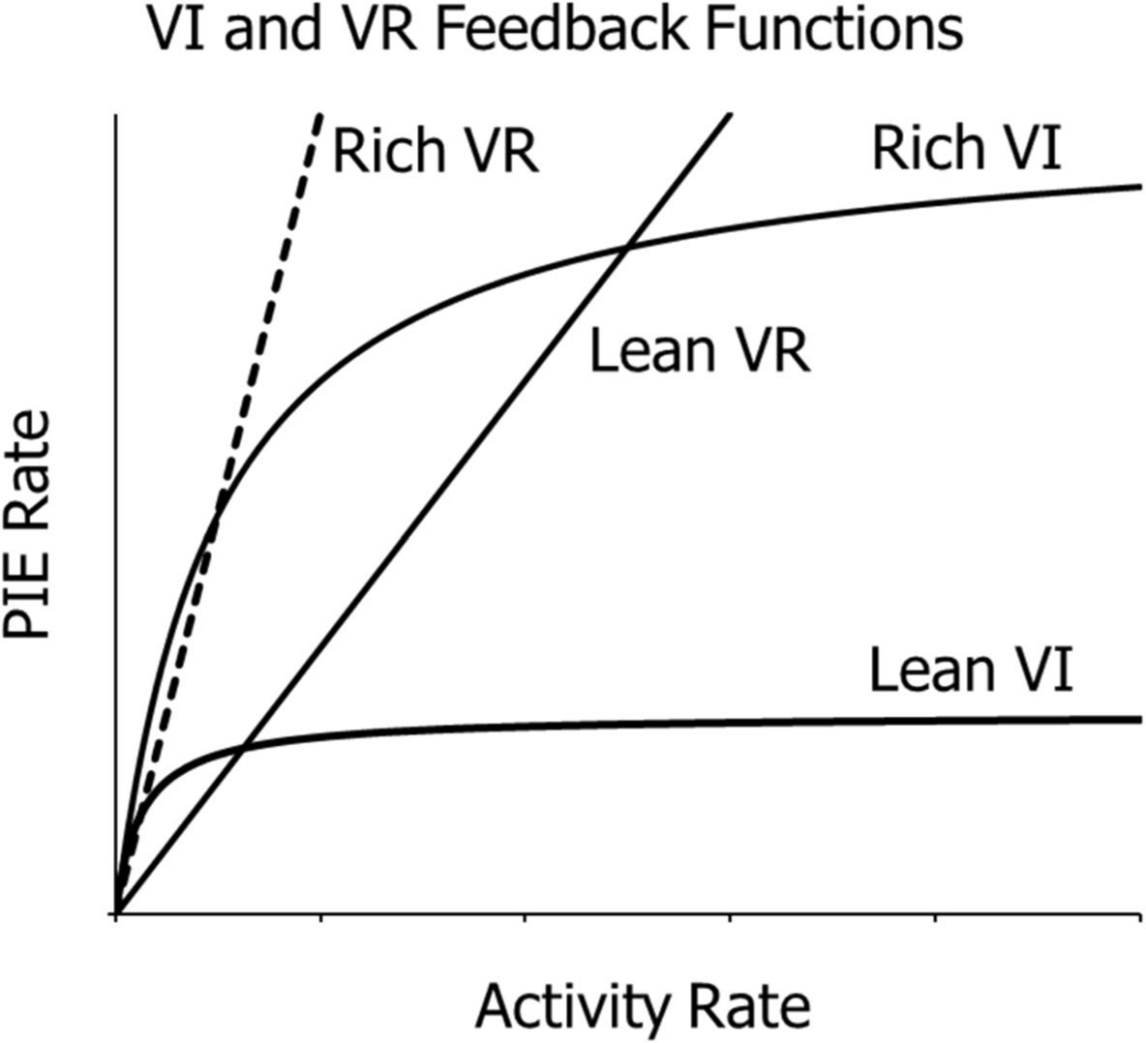
VI and VR Feedback Functions Showing How Rich VI Schedules become Ratio-Like. *Note.* The lean VI feedback curve only converges on the rich VR line at low activity rates, but the rich VI feedback curve overlaps with the rich VR line substantially

**Fig. 10:**
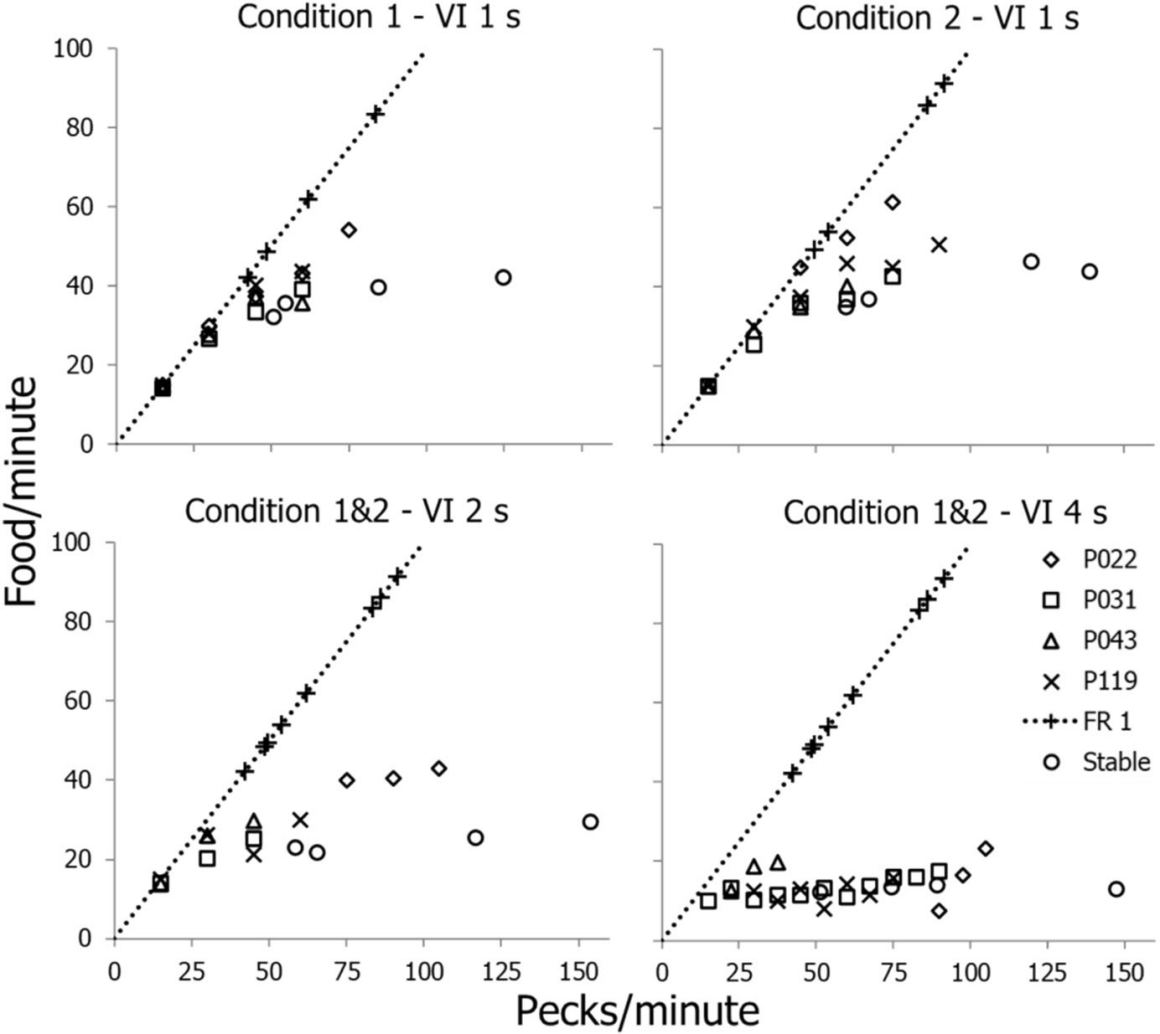
Empirically Derived Feedback Functions from the Experiment by Baum and Grace (2020). *Note.* Points are shown for the four pigeons. The dotted line depicts the FR 1 feedback line. Circles show stable performances

Baum and Grace (2020) dealt with the VI upturn extensively, so here I will only deal with it briefly. Figure 9 gives a basic understanding of how we explained the upturn. Figure 9 shows four feedback functions: for rich and lean VI and rich and lean VR. All four functions converge at the low end toward zero. The dashed line represents a rich VR, and the lower-sloped solid line represents a relatively lean VR. The lean VI remains distinct from both VR lines, but the rich VI curve overlaps with the rich VR line at lower peck rates. The richer the VI, the more it resembles a rich VR schedule. Rich VI schedules resemble a VR schedule across a substantial portion of the range of low peck rates. Thus, the reason VI peck rate grows as food rate gets high is that the VI schedule comes to function more and more like a VR schedule. Figure 10 shows this overlap for small VI schedules: VI 1 s, VI 2 s, and VI 4 s.

Figure 10 shows empirically derived feedback functions from the study by Baum and Grace (2020). The dotted line with crosses in each graph shows the feedback line and performances with FR 1. The two upper graphs show VI 1-s feedback functions derived empirically from the peck rates within components. The curves join the FR-1 line at a fairly high peck rate—about 40 pecks/min. The lower left graph shows feedback functions for FR 1 and VI 2 s. The VI feedback function joins the FR-1 line at a lower peck rate—about 20/min. The lower right graph shows the FR-1 line and the feedback function for VI 4 s, and the VI feedback function meets the FR-1 line at a relatively low peck rate—about 10 pecks/min. Relevant to the correction *d* in Eq. 8, the VI feedback functions fall short of the asymptotes that one would expect just from the schedule parameter. For VI 1 s, the curves fall short of 60 food/min (except, perhaps, for P022), because the delay *d* forces a lower food rate. Instead of 60 food/min, the asymptotic food rate equals $\frac{60}{t+d}$ where *t* equals 1 s and *d* equals about 0.5 s. In contrast, the points for VI 2 s fall fairly close to the predicted 30 food/min, and the points for VI 4 s fall on 15 food/min. The correction only affected the richest VI schedules.

Figures 9 and 10 provide a qualitative explanation for the VI upturn. Baum and Grace (2020) offered a quantitative treatment as well. Thus, because the low-range VI rates are explained by Eq. 6, the present focus will be on Phenomena 2 and 3 in Fig. 6: the canonical difference between VI and VR peck rates for the same food rate and the VR cutoff at low food rates known as “ratio strain.”

## *K*: A Matter of Units

If the measure of activities were time taken by each activity, as in Eq. 1a, *K* would equal the sum of the times—the denominator on the left side of Eq. 1a. The usual measure of time taken by an activity, however, is the number of switch operations generated by the interaction of the activity with the key, lever, or button. Thus, the units of *B* in Eq. 1b and Eq. 4 are switch operations. As in Eqs. 5 and 6, *K* is typically in units of *B*_*1*_-switch operations, ones generated by the operant activity.

Each switch operation represents a unit of time (Baum, 1976; Baum & Rachlin, 1969). Rarely does one know exactly how much time each unit represents, but one may infer the activity time by considering that the time is equal to the number of switch operations multiplied by the unit (*U* hereafter). Because all activities in Eq. 4 are measured in units of switch operations of the operant activity *B*_*1*_, *K* and *B*_*1*_ have the same units, and because the activities in the left-hand denominator of Eq. 4 take up all the available time, *K* is equal to 1/*U*. For example, if *U* equaled 0.01 s per switch operation, then *K* would equal 100 s. If *B*_*1*_ took up half the time available, *B*_*1*_ would equal 50. The fraction *B*/*K* equals the proportion of time taken up by *B*.

Different activities’ units result in different values of *K*. For example, McSweeney (1978) compared key pecking with treadle pressing in pigeons, presenting each activity by itself and also both activities concurrently. The estimates of *K* for treadle pressing were lower than those for key pecking, implying that the unit *U* for treadle pressing (time per switch operation) exceeded that for key pecking. When the two activities were presented concurrently, multiplying the food ratio by the ratio of *K* estimates accommodated the difference in *K* and resulted in typical matching of behavior ratio to food ratio (Baum, 1974, 1979).

A well-known difference between performance on VI and VR schedules is the difference in the manner of interacting with the response key. Pigeons exposed to VI food schedules “peck” at the key, reliably moving their head back and forth (Pear, 1985) and executing a series of smaller parts within the peck (Smith, 1974). Pigeons exposed to VR food schedules exhibit a different pattern, which Palya (1992) called “flicking,” and others have called “swiping” or “nibbling,” and these varied appellations probably arise from individual differences among pigeons. These activities all have in common that they result in higher switch-operation rates than “pecking.” Thus, each switch operation in VR activity represents a smaller amount of time than “pecking” in VI activity. As a result, *K* is larger for VR activity than for VI activity.

## The Behavior–Environment Feedback System

**Fig. 11:**
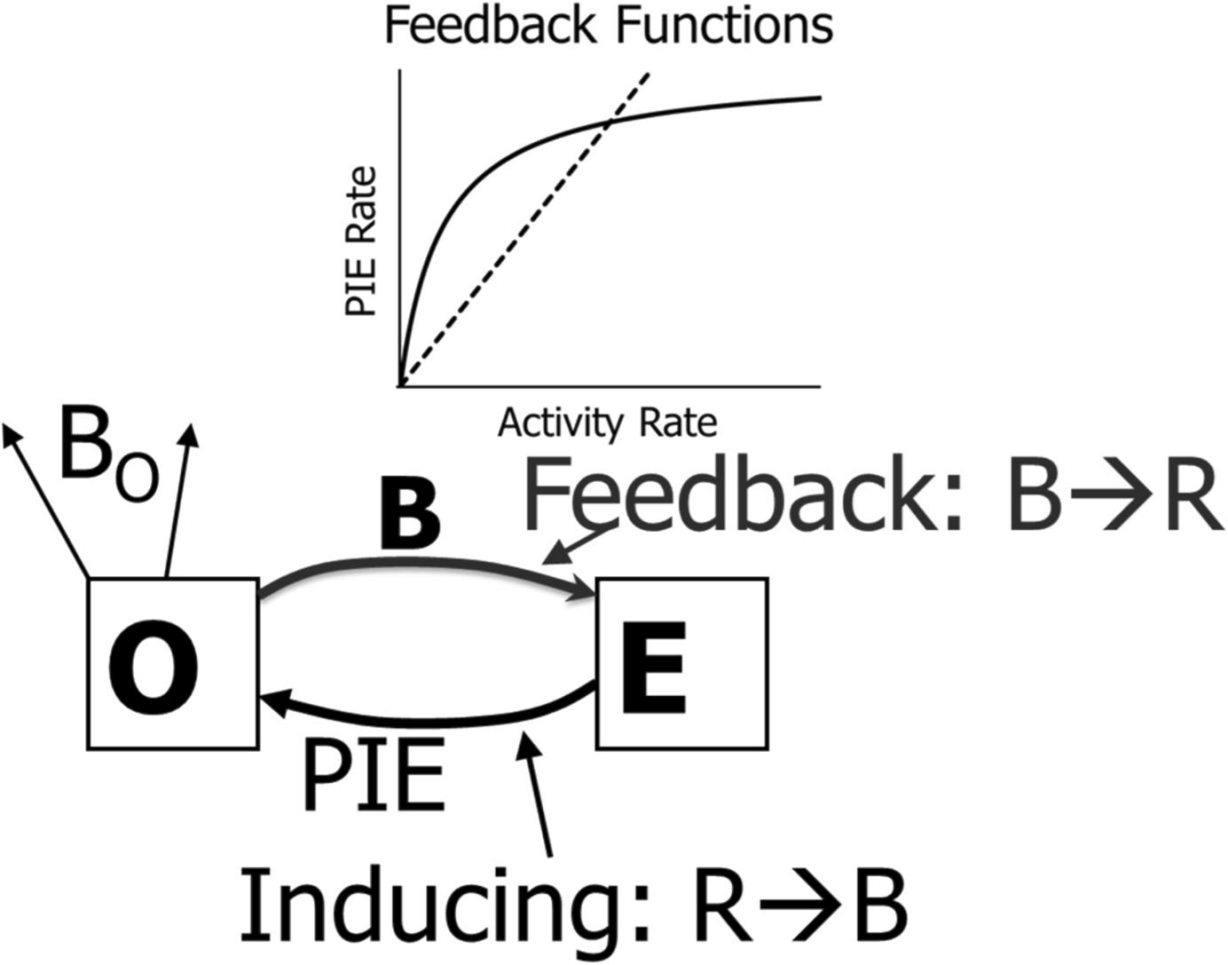
A Simple Depiction of the Behavior–Environment Feedback System. *Note.* The environment (E) affords a PIE that impinges on the organism (O), inducing both nonoperant activities *B*_*0*_ and operant activity *B* (R → B; food rate *R* induces activity rate *B*). The operant activity feeds back to E (B → R; activity rate *B* produces food rate *R*) according to either a VR feedback line (dashed) or a VI feedback curve (shown above)

According to the molar view of behavior, the environment and operant activity together constitute a feedback system, because operant activity produces PIEs that induce the operant activity that produces the PIEs, in a feedback loop (Baum, 1973, 1981, 1989). Figure 11 shows the basic concept in its simplest form. The environment (E) affords a PIE that impinges on the organism (O), inducing operant activity *B* and also nonoperant activity *B*_*0*_ that potentially competes with *B* (Baum & Aparicio, 2020). The bottom arrow indicates induction, and the top arrow indicates feedback, which follows either the feedback line for VR or the feedback curve for VI. The two processes, induction and feedback, constitute a closed-loop feedback system.

Figure 11 may clarify a key difference between VR and VI schedules. If the feedback PIE rate is governed by a rich VR, it may induce a higher operant rate, which would produce a higher PIE rate, and the system might run away to higher and higher rates, until it reached a physical maximum or competition from *B*_*0*_ and *B*_*N*_ (Eq. 4). On the other hand, if feedback PIE rate is governed by a lean VR (line d in Fig. 1), the PIE rate might be insufficient to induce that level of operant activity, which would lower the operant activity, decreasing the PIE rate further, and the system would drive rates down to zero. In contrast, if the PIE feedback rate is governed by a VI curve, it will drive operant activity up at low levels but not at high levels, as shown by the derivative in Fig. 2. Somewhere in between, the rates will come into an equilibrium in which the PIE rate produced just matches the PIE rate required for the maintained operant rate.

**Fig. 12:**
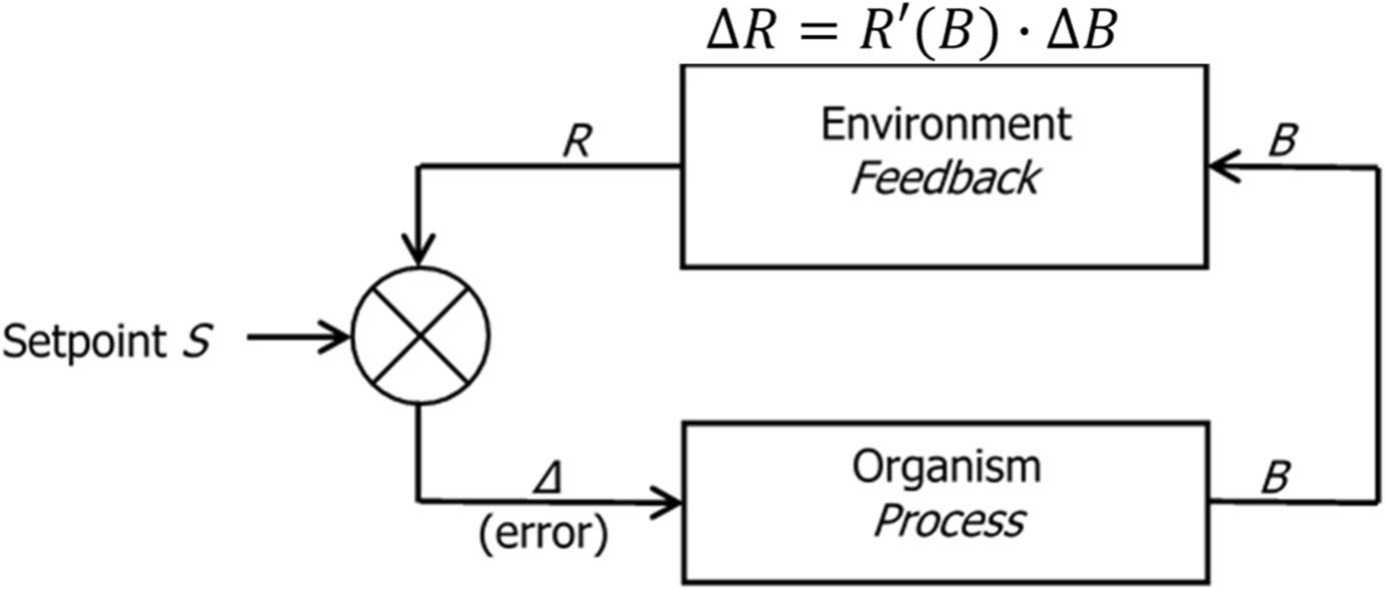
A Generic Behavior–Environment Feedback System. *Note.* The organism’s activity *B* is transformed by the environment into a PIE (food) rate *R*, which changes (ΔR) according to the derivative of *R* with respect to *B*, R’(B), if activity rate changes (ΔB). *R* is compared with a setpoint *S*. If error *Δ* differs from zero, induced activity *B* changes

Figure 12 shows a more formal representation of a generic behavior-environment feedback system. The organism process generates operant activity *B*, which is input to the environment process that generates *R* (PIE rate). Change in *R* (*ΔR*) depends on change in *B* (*ΔB*) according to the derivative $\frac{dR(B)}{dB}$, denoted *R’*(*B*) in Fig. 12. The equation above the feedback rectangle is Eq. 7 and provides the mechanism by which food rate *R* adjusts. *R* is compared with a standard or setpoint *S*, and the difference between *R* and *S* equals *Δ* (called “error”), which is input to the organism process and may change *B*. If the setpoint is attainable, error becomes zero, and the system stabilizes.

**Fig. 13:**
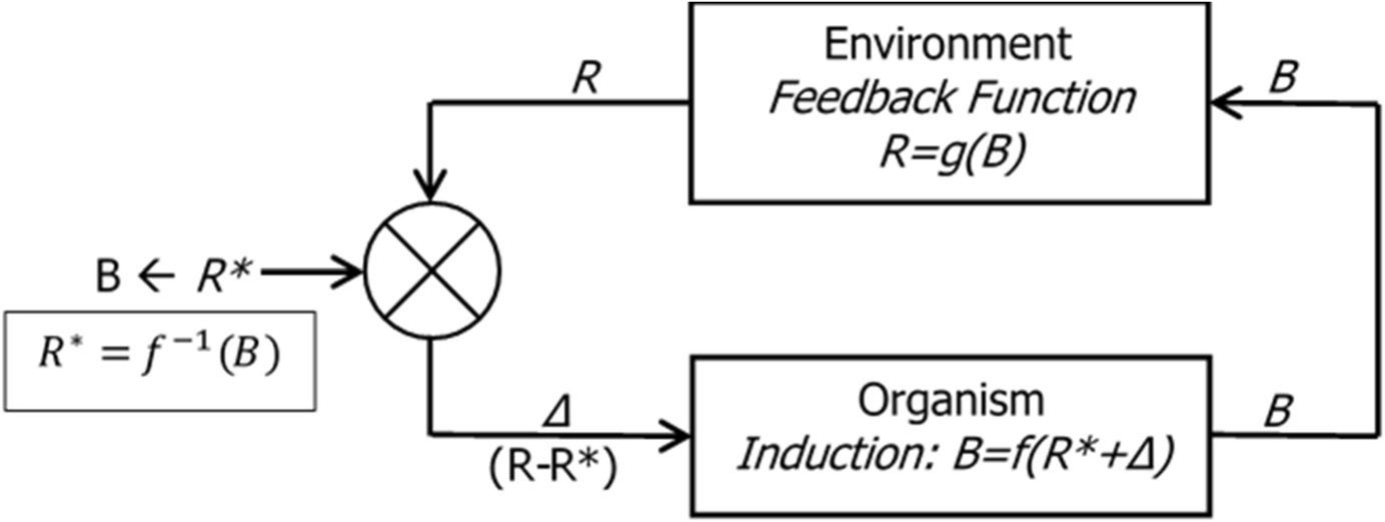
A Behavior–Environment Feedback System. *Note.* This would match feedback *R* to induction R*, but omits competition among activities

Figure 13 shows a feedback system that approaches the model we require. The organism process is induction of operant activity *B* according to a function *f*. The activity *B* is transformed into a PIE rate *R* according to a feedback function *g*. *R* is compared with R*, which depends on the present *B*; R*, as shown in the box, is the inverse function of *B*, *f*^*−1*^; it is the PIE rate that would induce *B*. The error *Δ* equals the difference *R*-R*, and is input to the induction process. If *Δ* is positive, and *R* is greater than R*, activity *B* increases. If *Δ* is negative, and *R* is less than R*, *B* decreases. This model is incomplete, because it omits competition among activities, as depicted in Fig. 11 and expressed in Eqs. 1a, 1b, and 4.

**Fig. 14:**
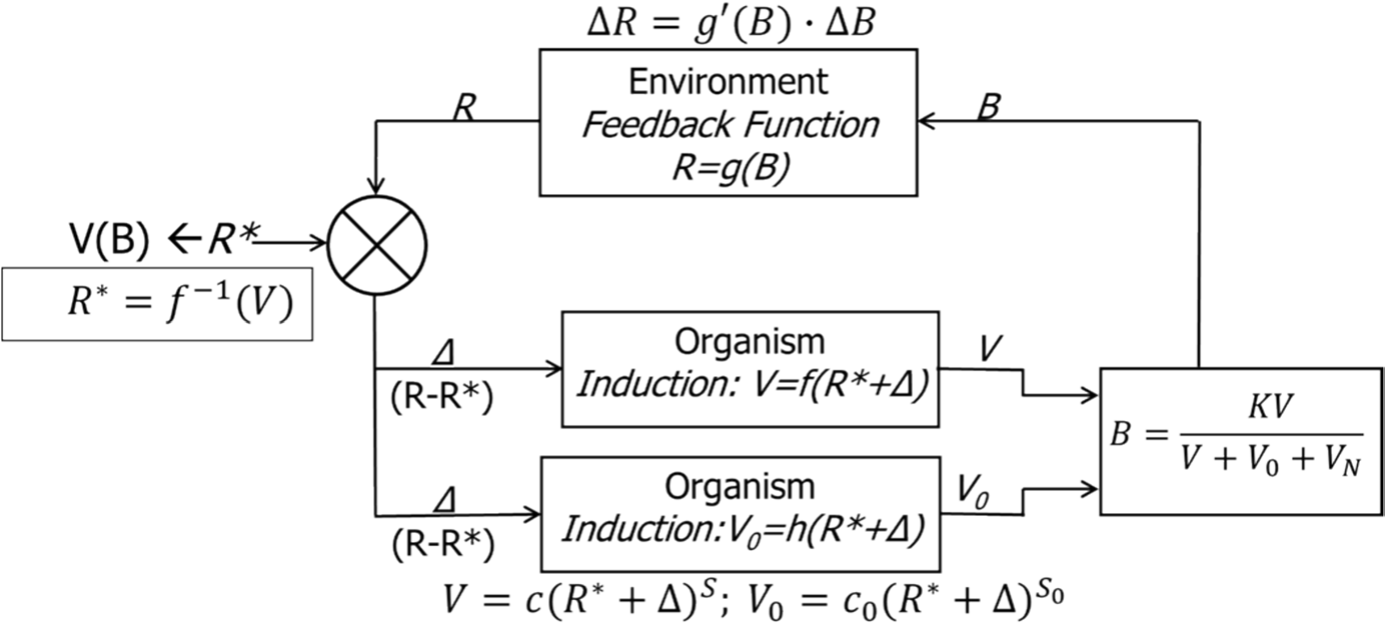
The Full Model for Explaining VI and VR Performance. *Note.* Competitive weight V depends on food rate according to a power function (Eq. 3). Error Δ is equal to the difference between food rate produced (R) and food rate required to induce competitive weight V. Error is input to two processes: induction of V and induction of V0. Activity rate B results from competition among *V*, *V*_*0*_, and *V*_*N*_

Figure 14 shows the full model we require, including not only *B* and *V* but also *B*_*0*_ and *V*_*0*_ and *B*_*N*_ and *V*_*N*_. Error results in induction of *V* according to function *f*, assumed to be a power function, and error results also in induction of *V*_*0*_ according to a function *h*, also assumed to be a power function. Operant activity *B* is the outcome of the interaction of *V* with *V*_*0*_ and *V*_*N*_. As in Fig. 12, the equation above the feedback process is a version of Eq. 7 with the derivative (slope) $\frac{dR(B)}{dB}$ or $\frac{dg(B)}{dB}$ denoted *g’(B)* and provides the mechanism by which *R* adjusts. R* is an inverse function *f*^*−1*^ of *V*, and equals the PIE rate that would induce the current level of competitive weight *V*. *V* and *V*_*0*_ are inputs to the process on the right, which, according to Eq. 4, gives *B* as output.

## *B*_*N*_: Activity Unrelated to the PIE

The full model shown in Fig. 14 has to include, not only *B*, *V*, *B*_*0*_, and *V*_*0*_, but also *B*_*N*_ and *V*_*N*_, because when food rate is low, *B* and *B*_*0*_ are low also, and *B*_*N*_ expands to be potentially significant, because *V*_*N*_ competes with *V* and *V*_*0*_, as in Eq. 4. Baum and Davison (2014) modeled *V*_*N*_ (they called it “r_N_”) as an interval-like relation, on the reasoning that *B*_*N*_ would consist of activities that arise from time to time, like grooming, stretching, or resting. The relation between *V*_*N*_ and *B*_*N*_ would be:

9 $${V}_{N}=\frac{1}{t+\frac{1}{{B}_{N}}},$$

where Baum and Davison (2014) estimated the parameter *t* to equal about 3.0. The estimate of *t* would presumably vary according to the several factors (e.g. deprivation level), but the situation we are considering here is comparable to the sort that Baum and Davison were modeling, so *t* is set here to 3.0.

When Eqs. 4 and 9 are taken together, one can set the denominator on the left side of Eq. 4 (*B* + *B*_*0*_ + *B*_*N*_) equal to *K* and substitute Eq. 9 for *V*_*N*_. A solvable equation results which allows calculation of *B*_*N*_:

10 $${B}_{N}=\frac{K-(V+{V}_{0})}{t\left(V+{V}_{0}\right)+1}.$$

When *r* equals zero, *V* and *V*_*0*_ equal zero, and *B*_*N*_ equals *K*; *B*_*N*_ takes up all the time. As *r* increases, *V* and *V*_*0*_ increase, and *B*_*N*_ decreases. When *V* + *V*_*0*_ equals *K*, *B*_*N*_ equals zero. Entering *B*_*N*_ into Eq. 9 gives a value for *V*_*N*_. Along with power functions for *V* and *V*_*0*_ and an estimate of *K*, the calculation of *V*_*N*_ allows *B* to be computed, as shown in Fig. 14.

## VR Performance

**Fig. 15:**
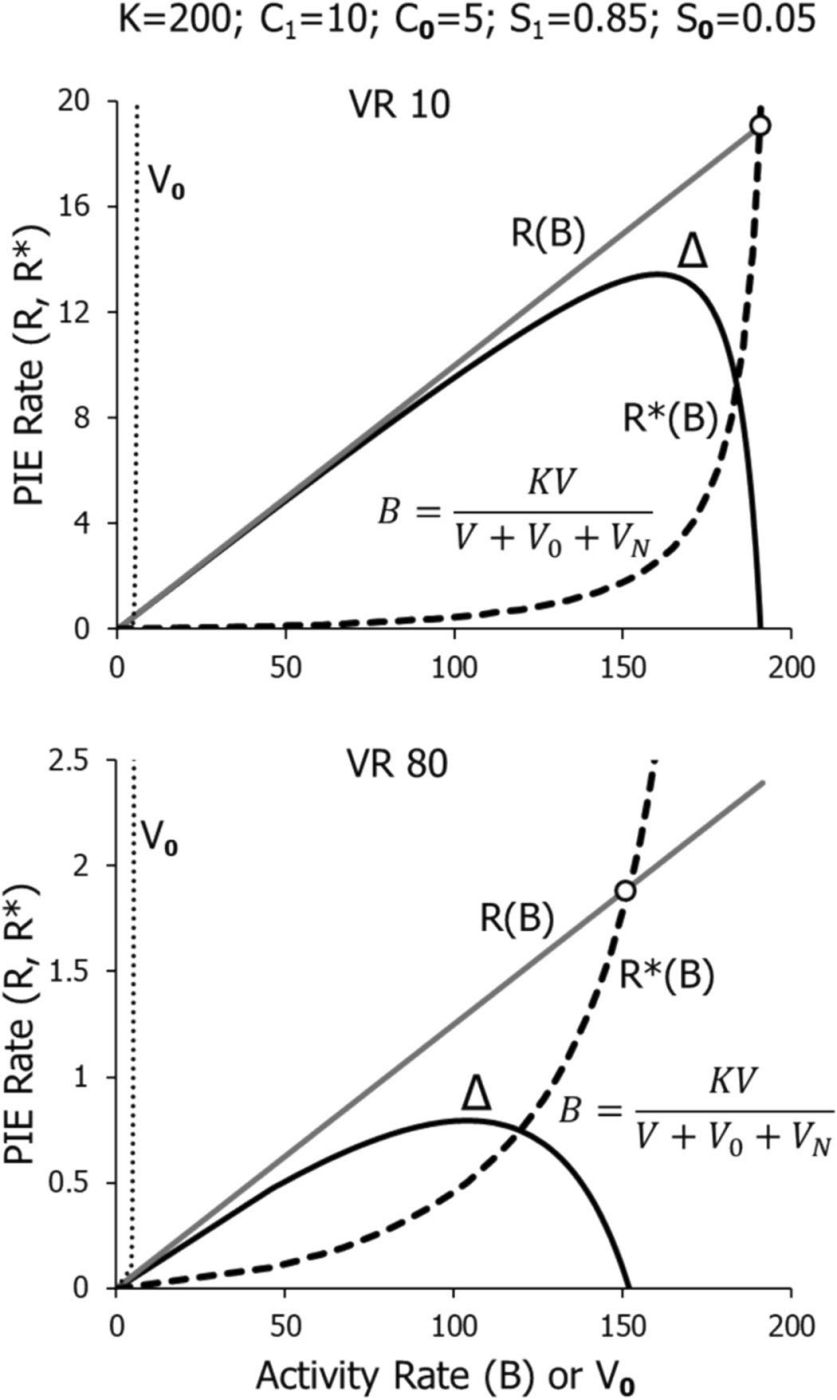
The Full Model Illustrated in Fig. 14 Applied to VR 10 and VR 80. *Note*. Where the error *Δ* hits zero, an equilibrium is reached, shown by the circle. Note different vertical axes

Figure 15 shows the model applied to VR 10 and VR 80. The gray line represents the linear feedback function. The dotted line shows *V*_*0*_, which varies little as PIE rate increases. The dashed curve represents Eq. 4, as shown; it plots R* on the vertical axis versus *B* on the horizontal axis. The solid curve shows *Δ* as a function of *B*. As *B* increases from zero, *Δ* increases at first and then decreases, reaching zero at a high activity rate. The circle shows the coordinates of an equilibrium point. It is an equilibrium point because greater activity rate causes PIE rate produced by the feedback function, *R*, to fall short of R*, the PIE rate necessary to sustain that level of *B*, making *Δ* negative and sending *B* back toward the equilibrium rate. If *B* falls short of the equilibrium point, *Δ* is positive, *R* exceeds R*, increasing *B* again. The equilibrium rate is lower for VR 80 than for VR 10, because *R* increases more slowly (lower sloped feedback function), causing *V* to grow more slowly relative to *V*_*0*_ and *V*_*N*_. The values of the parameters are shown at the top of Fig. 15. Only the ratio of *C*_*0*_ to *C*_*1*_ and the difference between *S*_*0*_ and *S*_*1*_ actually affect the outcome at high rates, as indicated in Eq. 6, although *V*_*N*_ may matter at low PIE rates.

**Fig. 16:**
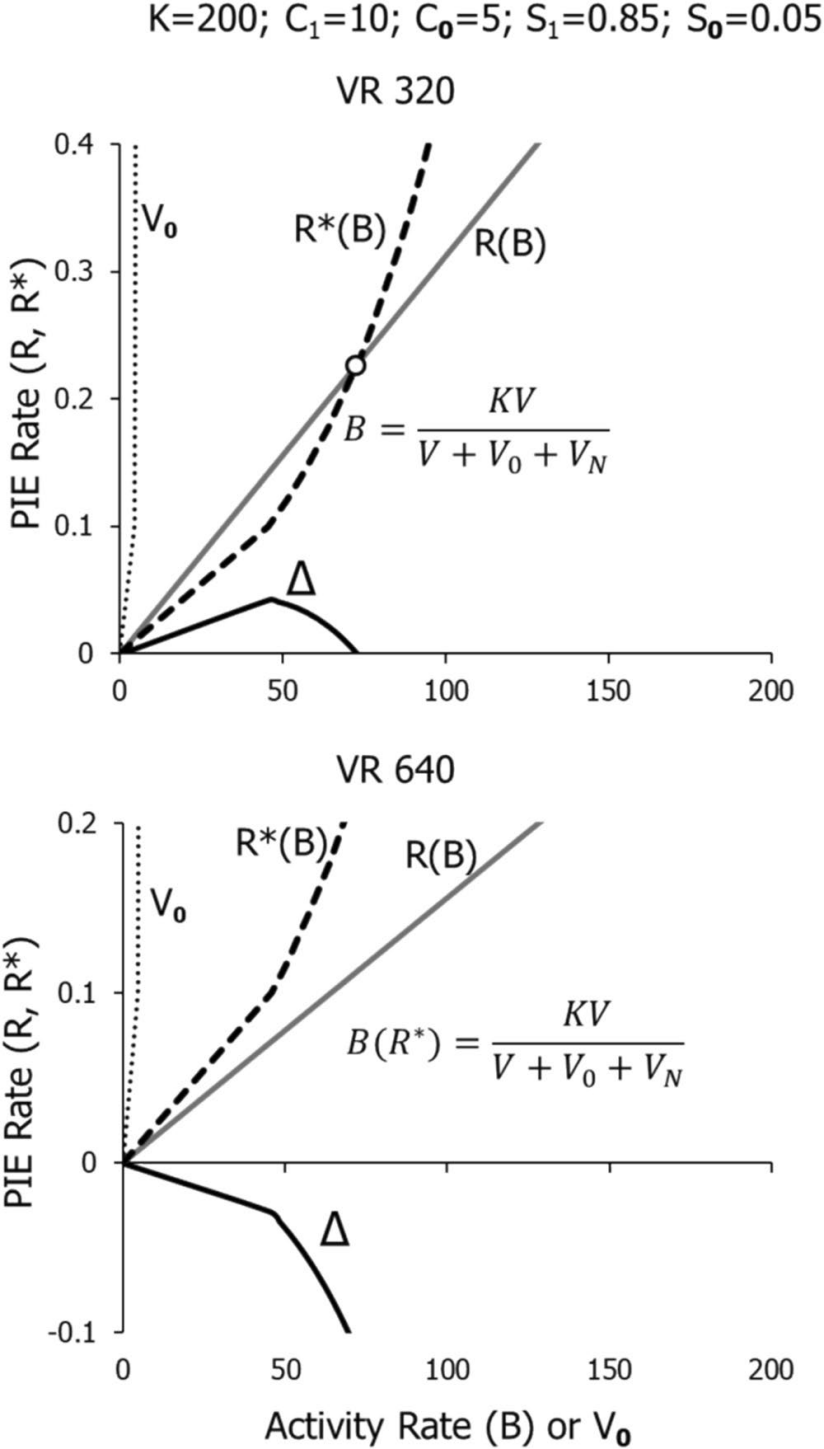
The Full Model Illustrated in Fig. 14 Applied to VR 320 and VR 640. *Note.* Error *Δ* falls to zero for VR 320, defining an equilibrium point (circle), but *Δ* is only negative for VR 640, indicating that VR 640 will maintain no activity. Note different vertical axes

Figure 16 shows the model applied to two large VR schedules, VR 320 and VR 640. The parameter values, shown at the top, are unchanged. Performance on VR 320 comes to an equilibrium point (circle) at a relatively low activity rate, because *Δ* reaches zero at about 70 with *K* equal to 200. With VR 640, however, *Δ* equals zero only at the origin, and as activity rate would increase, *Δ* would remain negative. Thus, VR 640 corresponds to line d in Fig. 1 and does not maintain any operant activity. The threshold of Fig. 1 lies between VR 320 and VR640.

## VI Performance

**Fig. 17:**
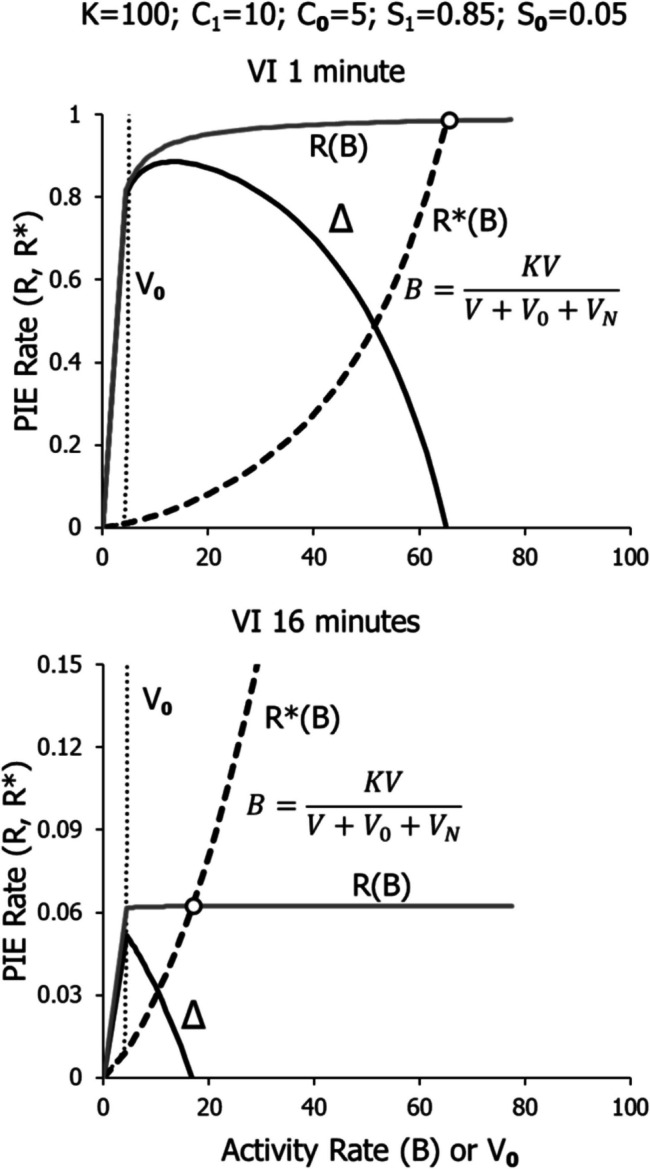
The Full Model Illustrated in Fig. 14 Applied to VI 1 Min and VI 16 Min. *Note.* Error drops to zero at the equilibrium point (circle). Note different vertical axes

Figure 17 shows the model applied to VI 1 min and VI 16 min. All the codes are as in Figs. 15 and 16, but the VR linear feedback function is replaced by the VI feedback curve. All parameters are the same as for VR, except *K* is set equal to 100, instead of 200. The equilibrium point (circle) occurs at a moderate activity rate for VI 1 and a relatively low rate for VI 16. As the VI schedule becomes leaner, the feedback function flattens out and will always intersect the dashed line representing R*, creating an equilibrium point, as in Fig. 1 (squares). No matter how lean the VI, it will maintain some activity. The basic reason for this is the change in slope of the feedback function with activity rate, as shown in Fig. 2.

**Fig. 18:**
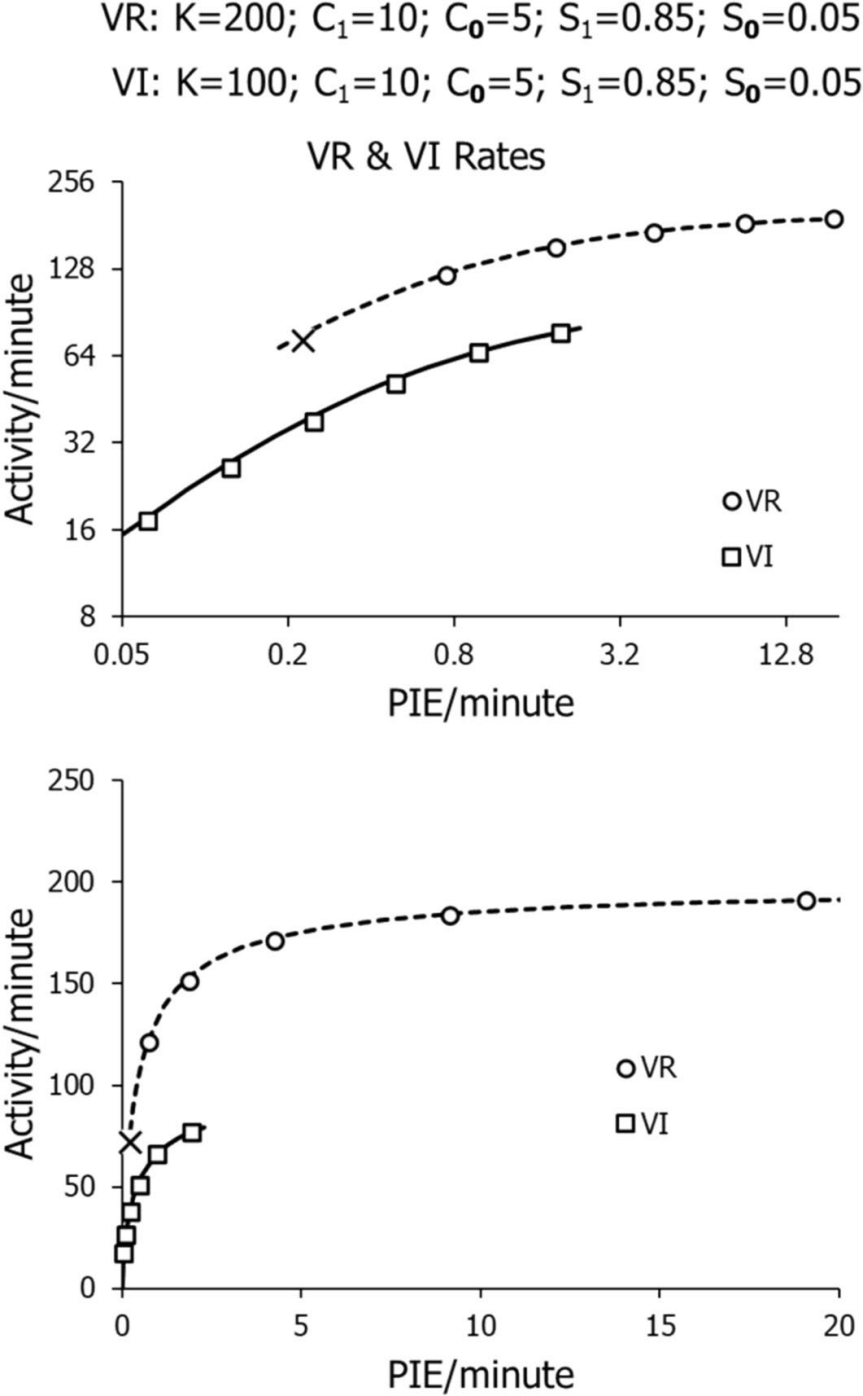
The Model Applied to Six VR and Six VI Schedules. *Note.* All points are equilibrium performances. The X shows performance on VR 320, close to the threshold below which a VR maintains no activity (“ratio strain”). The top graph shows the results in logarithmic coordinates. The bottom graph shows the results in arithmetic coordinates

Figure 18 shows the results of applying the model to 6 VR schedules and 6 VI schedules. All parameters are the same except that *K* is equal to 200 for the VR schedules and to 100 for the VI schedules. The circles show the equilibrium points for 5 VR schedules from VR 160 to VR 10. The X shows the equilibrium point for VR 320, close to the threshold VR (Fig. 1), below which no operant activity is maintained. The squares show the equilibrium points for VI 16 min to VI 0.5 min. The same results are displayed in logarithmic coordinates (top) and in arithmetic coordinates (bottom).

**Fig. 19:**
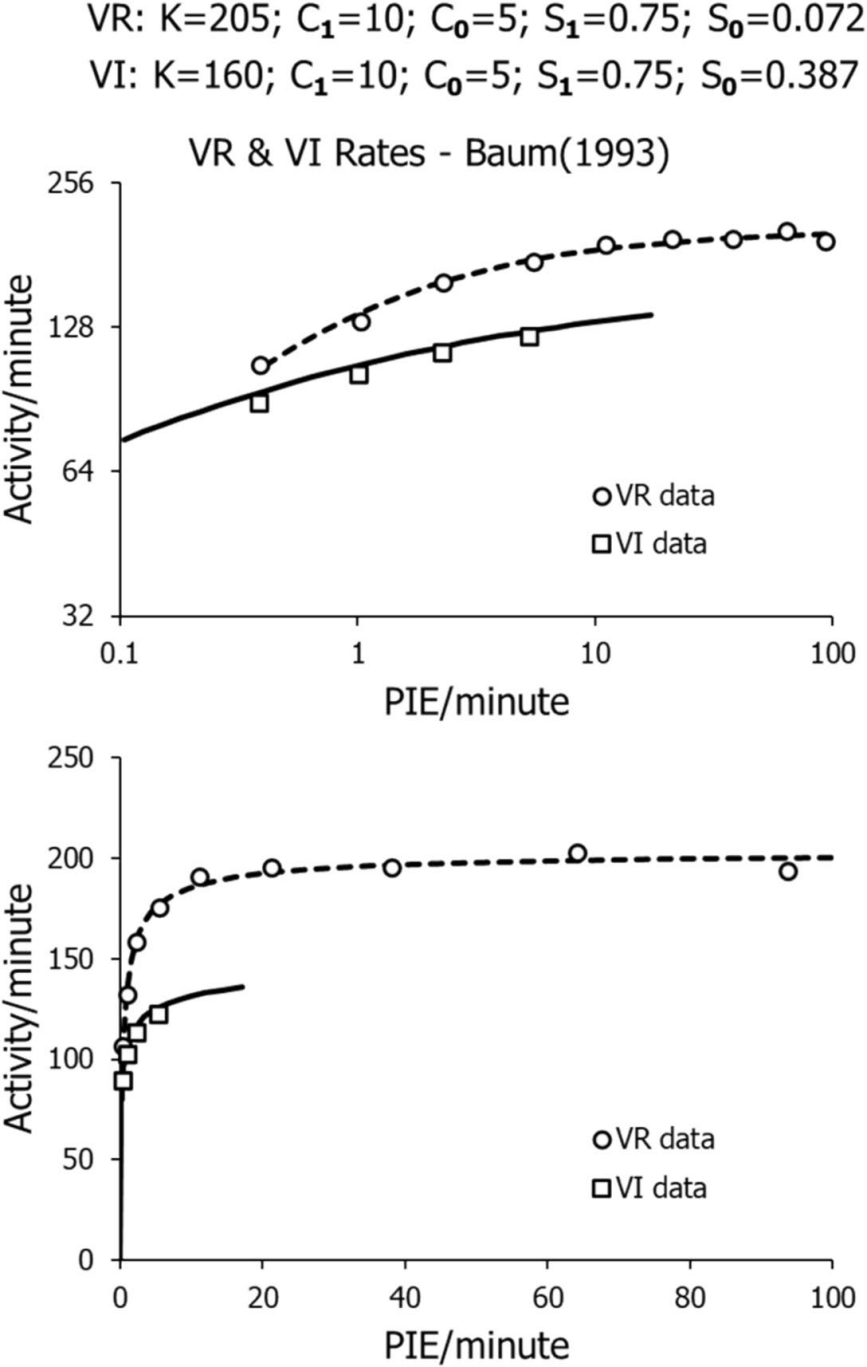
Activity Rates from Baum’s (1993) Study Fitted with Eq. 6. *Note.* Top graph shows fits in logarithmic coordinates. Bottom graph shows fits in arithmetic coordinates

Figure 19 shows the results of applying the model (Eq. 6) to the data shown in Fig. 4 (Baum, 1993): to the corrected peck rates maintained by the VR schedules (9 points) and just to the low-range VI rates (4 points). The parameters appear at the top. *K* was higher for the VR schedules, as expected. Due to the lesser curvature in the points for VI, *S*_*0*_ was set to a higher value (0.387) for VI than for VR (0.072) on the speculation that switching to *B*_*0*_ might be easier with a VI schedule than with a VR schedule. The difference in estimates might have diminished if more data were available at the lower VI food rates, as in Fig. 3. All other parameters were the same for VI and VR. The results are displayed in logarithmic coordinates (top) and arithmetic coordinates (bottom).

## Discussion

The model diagrammed in Fig. 14 produced results that match the differences in VI and VR performance with all parameters the same except for *K*, which differed because the activities induced by VR and VI schedules differ as to the time unit for each switch operation (*U*). When a pigeon flicks, swipes, or nibbles at the response key instead of pecking it, the time per switch operation (*U*) is less for VR than for VI, and *K* differs accordingly. Figure 18 shows that the model accounts for the key difference between VI and VR schedules sketched in Fig. 1: VR schedules cease to maintain operant activity when they become too lean, whereas VI schedules maintain operant activity no matter how lean.

Quantitative models are generally predictive, and this model is predictive, as seen in Fig. 18. The model predicts the shape of the performance curve relating activity rate to PIE (food) rate, and the model predicts the cutoff in VR activity at low food rates (the X). For any particular schedule, the point at which the feedback function intersects the model is the predicted stable performance, as shown in Figs. 15, 16, and 17.

The two curves in Fig. 18 differ only in *K*, representing the differing modes of interaction with the switch—key, lever, or button. The top graph shows that the two curves parallel one another in logarithmic coordinates. Were one to measure the time taken interacting with the switch, instead of counting switch operations, the two curves would superimpose. Although VI and VR performances overlap in the middle range of PIE rates, VI activity rates (solid curve) tend to be lower, and VR activity rates (dashed curve) tend to be higher. In the high range, extending the solid curve predicts that extremely short VI schedules can match fairly closely standard VR schedules in PIE rate produced. For example, Fig. 10 shows a VI 2 s to maintain about 23 food events per minute for a switch operation rate of 150 per minute or more, and the model predicts a VR 10 to maintain 18.4 PIE (food) per minute for a switch-operation rate of 184 per minute. The difference in *K* indicates that the two schedules maintain about the same time proportion taken by the operant activity.

In Fig. 19, where the model (Eq. 6) was applied with the parameters shown there, fitting the VI data required assuming a larger exponent for *B*_*0*_ than for the VR data. This was done because the difference between exponents for operant activity *B* and nonoperant activity *B*_*0*_ (*S* in Eq. 6) was larger for the VR schedules than for the VI schedules. To keep the exponent for the operant behavior the same across the two types of schedules, the difference was assigned to *B*_*0*_. Further research will determine whether this was a correct move. Because the experiment provided only 4 data points for VI, the fit to VI may be open to doubt. More points at lower food rates might have resulted in an estimate of *S* closer to that for VR. For example, in a study of VI peck rates as a function of contingent food rates (Baum, 2021), estimates of *S* across pigeons ranged from 0.641 to 1.05, with a mean of 0.773, comparable to the estimate for the VR schedules of 0.678 (0.75–0.072) shown in Fig. 19.

Induction following a power function is central to the model, as illustrated in Eqs. 2, 4 and 6. Such induction has considerable empirical support (Baum, 2020, 2021; Baum & Aparicio, 2020; Baum & Grace, 2020). Indeed, all reports of matching between two alternative activities provide at least indirect support, because the relation states that the ratio of activity rates equals the ratio of two power functions of food rate assumed to have the same exponent:

11 $$\frac{{B}_{1}}{{B}_{2}}=\frac{{{C}_{1}R}^{S}}{{{C}_{2}R}^{S}},$$

where the ratio of *C*_*1*_ to *C*_*2*_ is usually taken as the bias parameter *b* if *C*_*1*_ and *C*_*2*_ differ (Baum, 1979; Baum & Rachlin, 1969; Wearden & Burgess, 1982).

The model achieves what previous attempts failed to do: account for the differences between VI and VR schedules with the same simple model. As explained in the introduction, the failures usually resulted from researchers adopting a molecular view of behavior, in which behavior is conceived of as consisting of discrete events (“responses”), and relations between responses and inducers (“reinforcers”) are attributed to response-reinforcer contiguity or proximity. The present molar view succeeds because it relies on entities and relations extended in time: activities instead of responses, and feedback functions instead of momentary contiguity.

Whether the present model will generalize to other species and other activities remains to be seen. McSweeney (1978) found that a different activity, treadle pressing, produced results in pigeons similar to key pecking. The rates of treadle pressing as a function of food rate followed a pattern that could be fitted by Eq. 6, but with a low estimate of *K*, implying a larger time unit U. Considerable evidence supports power-function induction in rats (Baum, 2020; Baum & Aparicio, 2020; Baum et al., 2022). Norman and McSweeney (1978) produced data on VI schedules with rats in the lower range of food rates, and Soto et al. (2005) produced data with rats on VI schedules in the upper range, including the high-rate downturn. Data comparable to those analyzed here, within-subject parametric variation of VI and VR schedules over a wide range (e.g., Baum, 1993; Baum & Grace, 2020), seem to be absent for species other than pigeons.

## Conclusion

Adopting a molar view of behavior in combination with matching theory and power-function induction allowed explaining the different performances generated by VI and VR schedules. The simple model predicted the quantitative relation between activity rate and PIE (food) rate and the contrast between the cutoff for VR schedules at low rates versus the continued maintenance at low rates for VI schedules. The differences may be understood as the result of the difference in feedback functions for VI and VR schedules. The present theory treated only data from experiments with pigeons. Further research might explore the generality of the theoretical framework for other species.

## Data Availability

All data in this article are published and publicly available. Excel workbooks modeling data are available on request.
